# d-Xylose oxetane copolymers as bioderived and tuneable polyesters for amorphous solid dispersions†

**DOI:** 10.1039/d4lp00203b

**Published:** 2024-09-04

**Authors:** Ella F. Clark, Alexandra Howard, Sebastian D. Morales Feliu, James F. McCabe, Jonathan C. Burley, Vincenzo Taresco, Antoine Buchard

**Affiliations:** a Department of Chemistry, University of Bath Bath BA2 7AY UK; b University of Nottingham, School of Pharmacy Nottingham NG7 2RD UK; c Early Product Development and Manufacturing, Pharmaceutical Sciences, R&D AstraZeneca Macclesfield SK10 2NA UK; d University of Nottingham, School of Chemistry Nottingham NG7 2RD UK vincenzo.taresco@nottingham.ac.uk; e Department of Chemistry, University of York York YO10 5DD UK antoine.buchard@york.ac.uk

## Abstract

The ring-opening copolymerisation of cyclic anhydrides with an oxetane derived from natural monosaccharide d-xylose has been used to synthesise fully biobased water soluble polyesters, which are able to stabilise the amorphous phases of nifedipine and mefenamic acid, enhancing their apparent solubility in water up to 918 and 142% respectively. 2D picolitre-scale inkjet-printing, coupled with polarised optical microscopy (POM) analysis, enabled an initial, high-throughput miniaturised (ng–μg scale) screening of drug formulations. The best formulations were scaled up and analysed by FT-IR spectroscopy and DSC, revealing interactions between the drugs and polymers. Finally, drug dissolution studies demonstrated the effectiveness of the polymers in improving the drugs’ apparent solubility in water. These results showcase the potential of synthetic carbohydrate polymers as excipient for tailored drug formulations.

## Abstract 2

Biobased water soluble polyesters derived from xylose and made by ROCOP are able to stabilise the amorphous phases of nifedipine and mefenamic acid, enhancing their apparent solubility in water up to 918 and 142% respectively.

## Graphical abstract

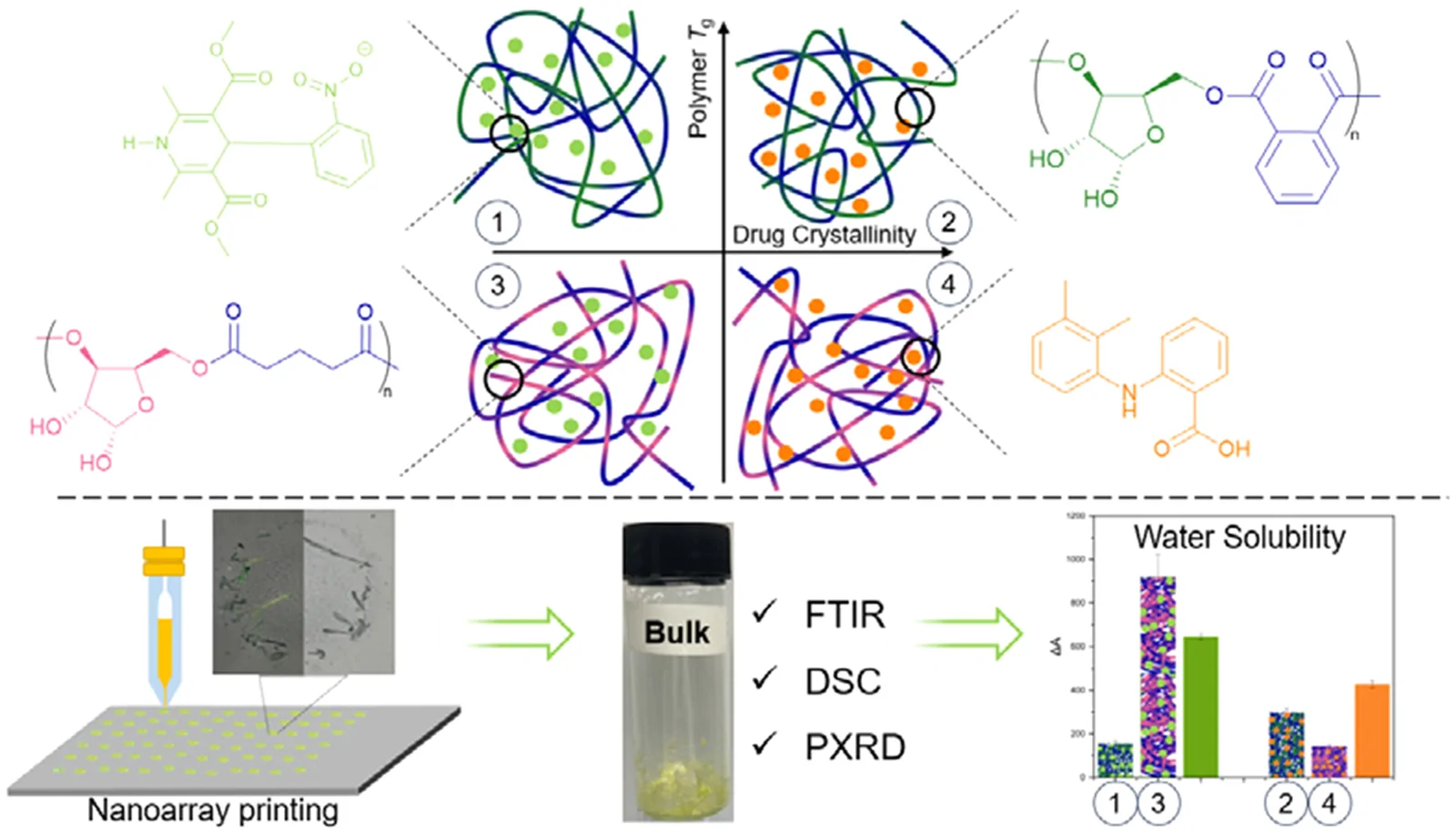

## Introduction

It is estimated that more than 70% of drug development candidates belong to class II, as defined by the Biopharmaceutics Classification System (BCS), where low solubility in water is the limiting factor in achieving a suitable bioavailability in patients.^1^ One common method of enhancing the apparent solubility of class II compounds is the amorphous solid dispersion (ASD) method, created when the active drug is molecularly dispersed in an inert, water-soluble polymeric matrix.^2,3^ This polymer matrix stabilises the drug in an amorphous state, lowering the thermodynamic barrier to dissolution, enhancing the apparent solubility. Strong interactions between the drug and polymer are key to preventing nucleation and crystallisation.^4^ However, no single polymer can effectively stabilise all amorphous drugs.^5–7^

To create an effective ASD, the polymer properties must be carefully considered to complement that of the drug. Miscibility with the drug, an amorphous nature and biocompatibility are only the start. Intermolecular interactions, such as hydrogen bonding, ionic or dipole–dipole interactions, result in strong interactions between the drug and polymer that may help to stabilise the amorphous state.^8,9^ Taylor and co-workers used FT-IR spectroscopy to show a good correlation between the hydrogen bonding ability of four different polymers (poly(vinyl alcohol) (PVA), poly(vinylpyrrolidone–vinyl acetate) (PVPVA), hydroxypropyl methylcellulose acetate succinate (HPMCAS) and poly(vinyl acetate) (PVAc)) and their effectiveness in inhibiting the crystallisation of hydrophobic felodipine.^10^ One indication of the intermolecular interactions between polymer chains is its glass transition temperature (*T*_g_) which is directly related to chain flexibility, steric hinderance, symmetry and polarity.^11^ The antiplasticisation effect refers to the increase in *T*_g_ of a drug, resulting from its combination with a high *T*_g_ polymer.^12^ Drug crystallisation is then inhibited as the molecular mobility becomes limited.^13,14^ However, the antiplasticisation effect is generally only effective to a point: a difference in *T*_g_ greater than 50 °C between the drug and polymer can conversely increase the molecular mobility of the drug and promote crystal growth.^15^ Therefore, it is not a universal solution to use a high *T*_g_ polymer to create an ASD. Finding polymers with a *T*_g_ up to 50 °C higher than the drug becomes a key step in determining which polymers are suitable for use in solid dispersions.

**Fig. 1:**
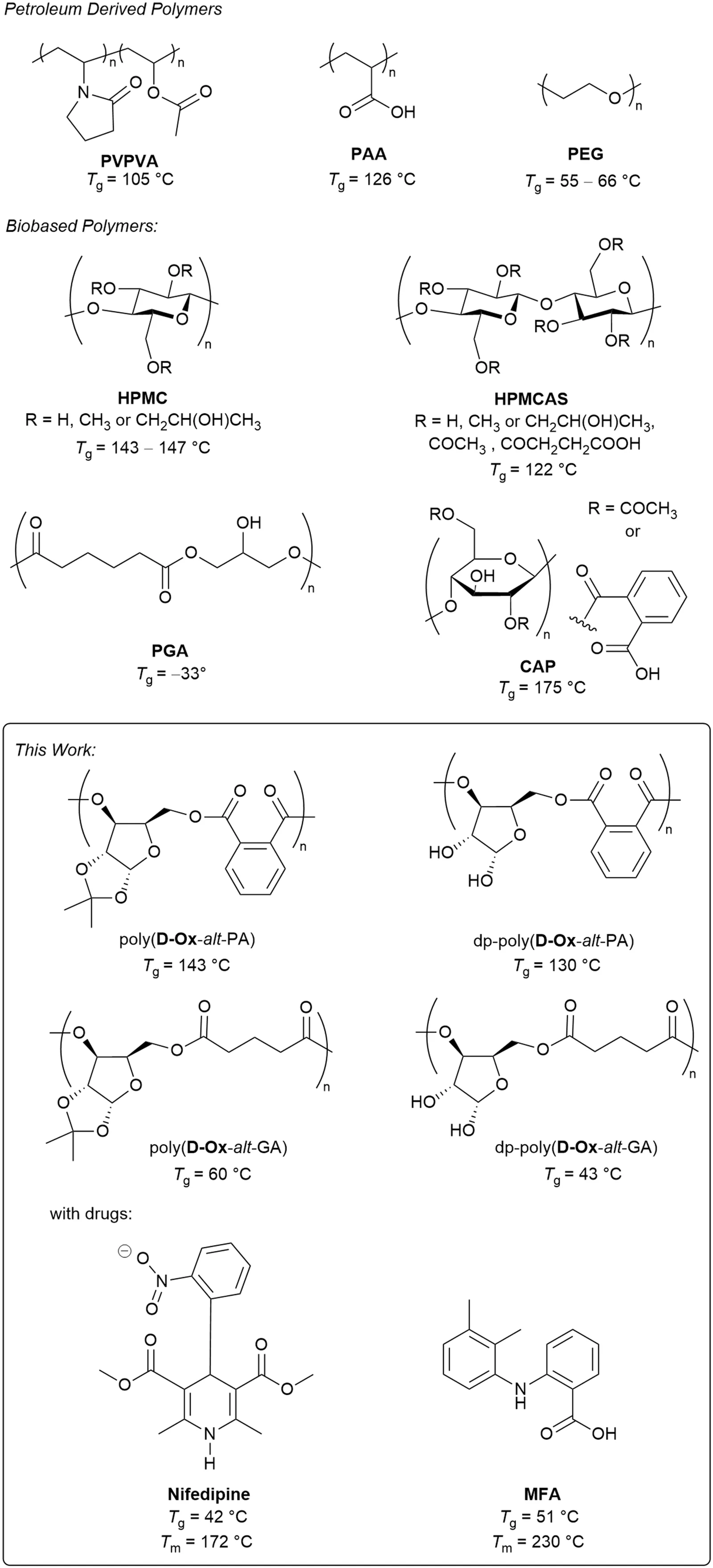
Top: Reported polymers, both petroleum (polyvinylpyrrolidone–vinyl acetate (PVPVA), polyacrylic acid (PAA) and polyvinylpyrrolidone (PVP)) and bioderived (hydroxypropyl methylcellulose (HPMC), hydroxypropyl methylcellulose acetate succinate (HPMCAS)), polyglycerol adipate (PGA) and cellulose acetate phthalate (CAP) that have been used for ASDs.^2,21^ Bottom: Sugar-derived polymers described herein for ASDs of nifedipine and mefenamic acid (MFA).

A limited number of polymers have been so far approved by the Food and Drug Administration, including polyethylene glycol (PEG), PVA and PVPVA, but these polymers do not provide sufficient structural diversity to enable a detailed structure activity relationship.^16–18^ With sustainability concerns in mind, cellulose-based polymers such as hydroxypropyl methylcellulose (HPMC) and HPMCAS, can be used in ASDs to replace petroleum derived, synthetic polymers such as PVP, and PVPVA (Fig. 1).^2,19,20^ Limited materials and time often pose challenges in the initial phases of drug development. Utilising high-throughput methods to assess drug crystallinity and solubility offer significant benefits in the rapid optimisation of ASDs, often mitigating substantial costs. Examples of high-throughput systems have been published which show good correlation to upscaled ASD results, although many were limited by their dependence on multiple dilution steps, no automated sample preparation and the necessity to premix the ASD solutions.^22–24^ Inkjet printing is a versatile, fully automated and economical technique, used to deposit liquids on a surface with high precision.^25,26^ It also allows for small batch screening on the nanogram scale which presents exciting opportunities for new drug delivery techniques.^27^

In parallel, the Ring-Opening Copolymerisation (ROCOP) of cyclic ethers/anhydrides provides a simple method for polyester synthesis.^28^ One major advantage of ROCOP is that the *T*_g_ and functionality of the resulting polyester can be controlled by variation of the cyclic anhydride. For example, switching the cyclic anhydride from phthalic anhydride (PA) to glutaric anhydride (GA) (which can both be biosourced),^29,30^ lowers the *T*_g_ of butylene oxide/allyl glycidyl ether copolymers from 38 to −39 °C respectively.^31^ Our group, among others, has also identified xylose as a rich sustainable feedstock for polymer synthesis, owing to their low cost, low toxicity, and high abundance.^32–34^ The presence of multiple hydroxy groups also offer the potential for pre- or post-polymerisation functionalisation.^35^ Previously, we have reported the ROCOP of a d-xylose 3,5-anhydrosugar derived oxetane, **d-Ox**, with seven cyclic anhydrides, producing a family of bioderived polyesters with *T*_g_s ranging from 60–145 °C.^36^ To the best of our knowledge, polyesters produced *via* ROCOP have not been reported for ASD formulation.

Herein, we report the synthesis of two fully bioderived water soluble polyesters, dp-poly(**d-Ox**-*alt*-PA) and dp-poly(**d-Ox**-*alt*-GA) (Table 1), *via* the ROCOP of **d-Ox** with PA and GA respectively, followed by acetal deprotection, and their formulation with mefenamic acid (MFA) and nifedipine, selected as representatives of class II BCS drugs (Fig. 1). The water insoluble polymer precursors, poly(**d-Ox**-*alt*-PA) and poly(**d-Ox**-*alt*-GA), have also been investigated to probe the influence of the polymers’ *T*_g_ and water solubility on their suitability for ASDs. 2D inkjet printing was first used to screen polymer–drug combinations on the nanogram scale, followed by more detailed analysis of the upscaled ASDs and drug dissolution studies.

**Table 1:** Post-polymerisation acetal deprotection to yield water-soluble hydroxy-functionalised polymers

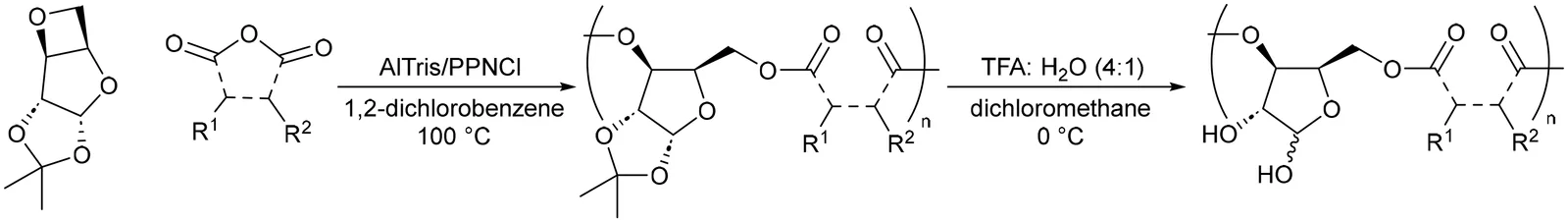					
Polymer	*M*_n, SEC_ a	*Đ*_M_ b	*T*_g_ c	*T*_m_	Water soluble
Poly(**d-Ox**-*alt*-PA)	11 300	1.20	143	—	No
dp-Poly(**d-Ox**-*alt*-PA)	10 100	1.29	130	—	Yes
Poly(**d-Ox**-*alt*-GA)	6200	1.94	62	—	No
dp-Poly(**d-Ox**-*alt*-GA)	3000	2.18	43	—	Yes

a Calculated by SEC (against polystyrene standards in THF, or PEG standards in DMAc/LiBr for dp-polymers).

b *Đ*_M_ = *M*_w_/*M*_n_.

c Values taken from DSC second heating cycle.

## Results and discussion

### Polymers synthesis and optimisation

Previously we have reported the ROCOP of **d-Ox** with seven cyclic anhydrides, catalysed by *rac* 1,2-cyclohexanediamino-*N*,*N*′-bis-(3,5-di-*t*-butylsalicylidene)chromium(iii), CrSalen.^36^ The catalyst system was optimised, with three new complexes tested (Table S1†). An Al-centred aminotrisphenolate complex (AlTris) was found to provide enhanced activity while also yielding highly selectively high molar mass (*M*_n_) co-polymers. Using AlTris as the catalyst and bis(triphenylphosphine)iminium chloride (PPNCl) as the initiator (**d-Ox **: anhydride : AlTris : PPNCl ratio of 200 : 200 : 1 : 1), two **d-Ox** polyester copolymers were thus synthesised with PA and GA, with >99% conversion of **d-Ox** calculated by NMR analysis, yielding poly(**d-Ox**-*alt*-PA) and poly(**d-Ox**-*alt*-GA), respectively (Table 1).^36^ An *M*_n_ of 11 300 g mol^−1^ (*Đ*_M_ = 1.20) and 6200 g mol^−1^ (*Đ*_M_ = 1.94), were obtained for poly(**d-Ox**-*alt*-PA) and poly(**d-Ox**-*alt*-GA) respectively, as measured by SEC (in THF, against narrow polystyrene standards).

The *T*_g_s of these polymers were 143 °C and 62 °C respectively, as observed by DSC in the second heating cycle. Deprotection of the xylofuranose core acetal group was achieved *via* acid hydrolysis with trifluoroacetic acid (TFA), yielding two DMSO and water soluble polyesters: dp-poly(**d-Ox**-*alt*-PA) and dp-poly(**d-Ox**-*alt*-GA) (deprotected at >99% and 93%, respectively) (Table 1).^35^ SEC analysis (in DMAc/LiBr, against narrow PEG standards) showed the resulting polymers had a *M*_n_ of 10 100 g mol^−1^ (*Đ*_M_ = 1.29) and 3000 g mol^−1^ (*Đ*_M_ = 2.18) respectively, with a *T*_g_ of 130 and 43 °C. Those changes in *M*_n_ and dispersity may be due to changes in SEC method and in polymer hydrodynamic volumes, affected by the presence of OH groups. During deprotection, acid catalysed transesterification may also happen, even if no evidence of branching was seen by NMR spectroscopy. Polymer molar mass and dispersity have been shown to impact ASD performance,^37^ and the molar masses of the polymer studied here are relatively low and some dispersities high. However, it is worth noting that their thermal properties are in line with most commercial polymers used (usually at higher molar masses), including with HPMC (*vide infra* dissolution studies). In addition, having short polymer chains is likely to make ASD more (bio)degradable and therefore can be seen as an advantage.

The **d-Ox** copolymers can be fully bioderived, potentially providing a more sustainable alternative to petroleum derived polymers for ASDs such as PEG or PVA. This synthetic approach also allows for improved control and tuneability over chemically extracted polymers such as HPMC and HPMCAS. HPMC and HPMCAS have been used in solid dispersions but suffer from high melt viscosity, preventing them from being scaled to the industrial level using hot melt extrusion.^38,39^ They are also typically incompatible with lower *T*_g_ drug compounds.^40,41^ For example, nifedipine, with a *T*_g_ of 42 °C,^37^ falls below the 50 °C optimal range of most common bioderived polymers, which have a *T*_g_ > 92 °C (HPMC, HPMCAS) (Fig. 1). We hypothesised that the thermal properties of dp-poly(**d-Ox**-*alt*-GA) (*T*_g_ = 43 °C) would be well suited to produce nifedipine ASDs, with the acetal deprotection also providing H-bonding between the polymer and the drug. Moreover, dp-poly(**d-Ox**-*alt*-PA) would also be well matched to MFA (Fig. 1), another type II drug, which has been shown to be more challenging to stabilise than nifedipine, but also more compatible with high *T*_g_ polymers (although its *T*_g_ has been calculated to be 51 °C).^42,43^

### 2-D inkjet printing

Here, 2-D inkjet printing was used as an initial, high-throughput and nano-scale method to compare the behaviour of each of the polymers in ASDs. Bradley and co-workers first reported such a high-throughput 2D printing technique, printing microarrays of carbamazepine, sulfamethoxazole, and 2-[(2-nitrophenyl) amino]-3-thiophenecarbonitril onto different commercial polymers, and analysing them using Raman spectroscopy.^44^ This was possible with only 27 μg of polymer and 1 mg of drug. Since this pioneering work, the 2D inkjet printing of microarrays to screen polymer–drug solid dispersions has been expanded and optimised.^45,46^ Taresco and co-workers used a piezo electric inkjet printer to print PVPVA with six different drugs, showing 2D inkjet printing could be used to approximate the threshold for the drug loading in bulk and correctly correlate the microarray data to the literature.^45^

**Fig. 2:**
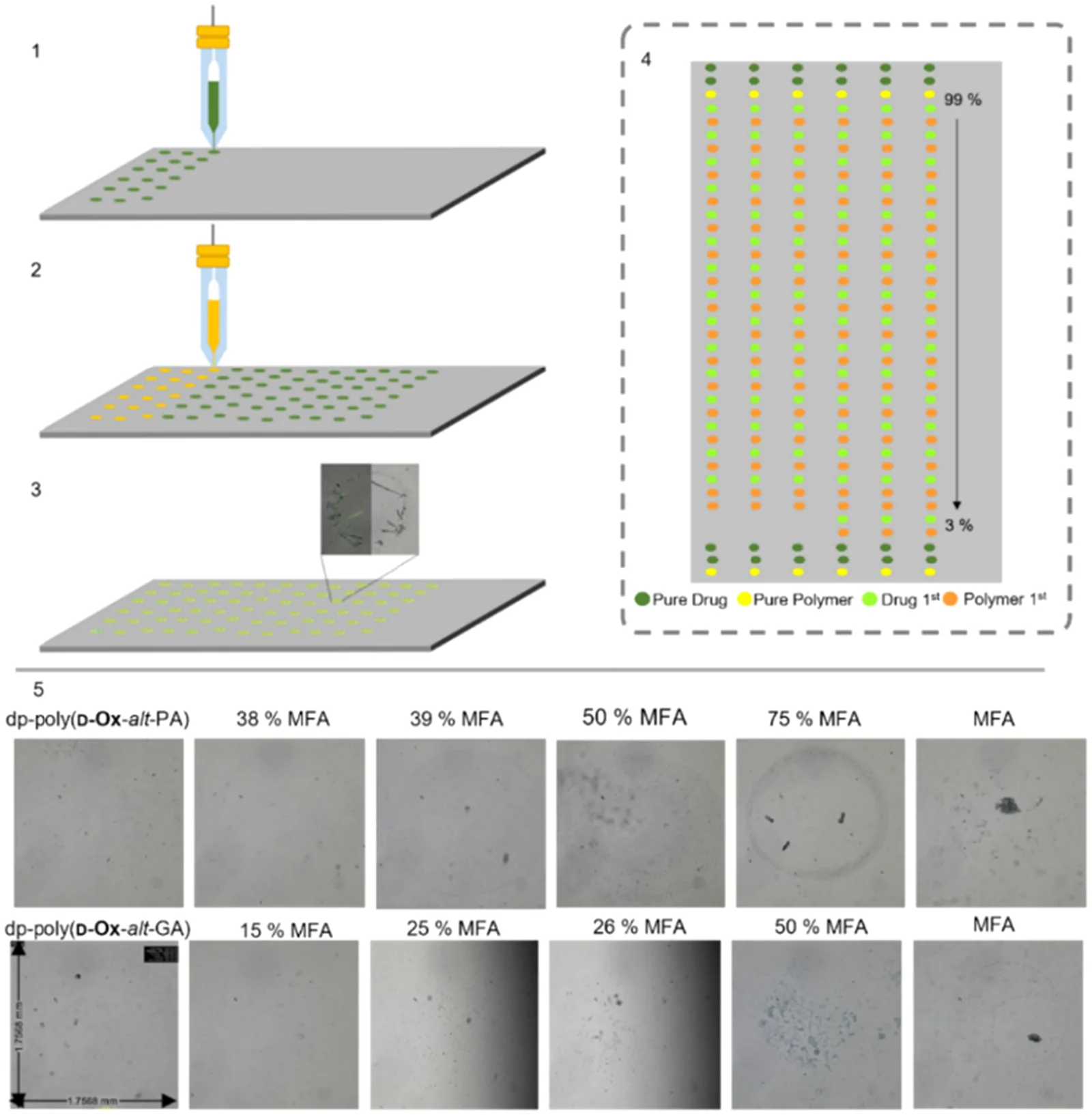
Schematic representation of the sequential steps needed to develop a solid microarray (1) Printing the drug. (2) Printing the polymer. (3) Solvent evaporation. (4) Schematic of the microarray, “%” reflect the weight/weight % ratio of MFA/polymer. (5) Optical microscopy images (48 hours after printing) of the dp-poly(**d-Ox**-*alt*-PA) and dp-poly(**d-Ox**-*alt*-GA)–MFA microarray varying the weight/weight ratio from 0–100%.

In the present work, six microarrays were printed: nifedipine or MFA with poly(**d-Ox**-*alt*-GA), dp-poly(**d-Ox**-*alt*-PA) or dp-poly(**d-Ox**-*alt*-GA). Poly(**d-Ox**-*alt*-PA) was not printed due to poor solubility in DMSO. Polymer solutions of 1 mg mL^−1^ and drug solutions of 5 mg mL^−1^ were printed with an average final droplet total volume of 314.6 pL. The weight/weight % ratio of drug/polymer was varied from 100–0%, with an average of 1.6 ng of drug per composition. To investigate if diffusion enabled sufficient mixing, each composition was printed in duplicate, first by printing the drug then the polymer at a specific spot, and second, in the adjacent column, by printing the polymer then the drug (Fig. 2). The slides were left for 48 hours at 50% relative humidity and 29.8 °C to allow DMSO to evaporate then analysed with non-polarised and polarised optical microscopy (POM).

Microscopy images of the pure excipients were either transparent/translucent (with dp-poly(**d-Ox**-*alt*-PA) and dp-poly(**d-Ox**-*alt*-GA)) or opaque (with poly(**d-Ox**-*alt*-GA)), and did not exhibit any birefringence, consistent with their amorphous character. For the pure drugs, POM showed that MFA formed birefringent crystals after two days, whereas nifedipine did not exhibit any obvious birefringence under crossed-polars, suggesting an amorphous character. Similar behaviour has been reported for a microarray of flufenamic acid when inkjet printed, and was attributed to the low volumes resulting in abnormal crystallisation behaviour.^45^

**Fig. 3:**
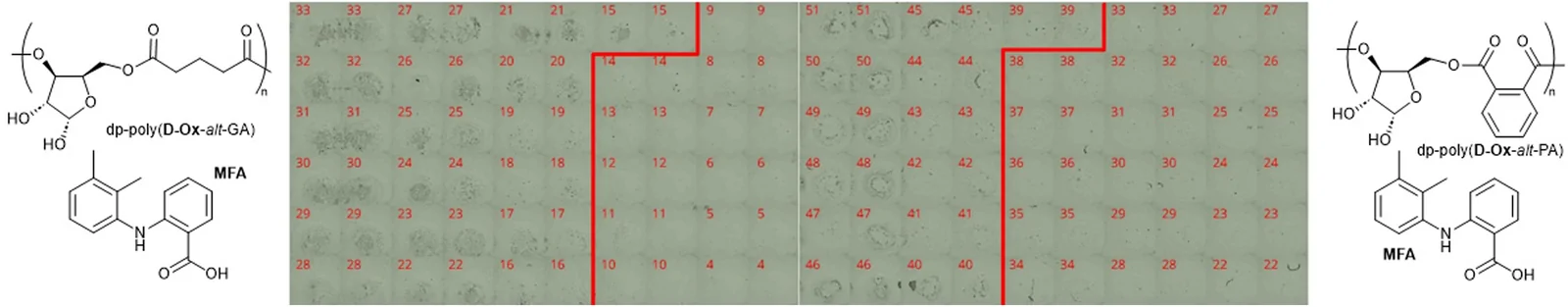
Phase contrast images (two months after printing) for % w/w combinations (as labelled) of MFA with: dp-poly(**d-Ox**-*alt*-GA) (left) and dp-poly(**d-Ox**-*alt*-PA) (right). Each individual image is *ca.* 1.75 mm across. Columns are in duplicate: the drug-first printed spots appear on the left and the excipient-first on the right. Numbers refer to the loading of MFA (% w/w). The red line denotes the estimated transition point where birefringence appears (as a function of increasing composition).

The order of printing was shown to not influence the occurrence of crystallisation. All nifedipine spots remained smooth and transparent, suggesting the dispersion remained amorphous after two days. Dp-poly(**d-Ox**-*alt*-PA/GA)–MFA spots suggested amorphous dispersions up to 38% and 25% w/w MFA loadings respectively (Fig. 3). Improved stability of MFA in dp-poly(**d-Ox**-*alt*-PA) may be due to non-covalent interactions between aromatic groups as MFA is less polar than nifedipine. Previously, PVAc has been reported to stabilise MFA up to a 50% w/w when printed, whereas PEG was only effective up to an MFA loading of 20% w/w and is therefore outperformed by dp-poly(**d-Ox**-*alt*-PA).^47,48^

Optical microscopy revealed aggregation occurred with poly(**d-Ox**-*alt*-GA) ASDs with nifedipine and MFA, with phase contrast showing MFA microcrystals (length < 61 μm) were evenly dispersed throughout the aggregated polymer up to a MFA loading of 51% w/w (Fig. S5†). Beyond 51% w/w, larger birefringent crystals were observed. This highlighted the necessity for polar, water-soluble polymers for those ASD formulations.

Microscopy images were subsequently collected for the micro-array samples after storage at room temperature for approximately two months to probe the ASDs stability (Fig. 3). The lack of birefringence of the pure excipients first indicated that the polymers were still amorphous. For all binary combinations of nifedipine with dp-poly(**d-Ox**-*alt*-PA/GA) or poly(**d-Ox**-*alt*-GA), at all compositions 0–100% w/w, the printed spots were transparent, translucent or opaque, without obvious structure in phase-contrast images, and did not exhibit any obvious birefringence under crossed-polars. This suggested that these samples were still amorphous, showing nifedipine had not crystallised during storage. This lack of crystallisation is in line with literature reports of nifedipine behaviour such as its class II glass-forming ability.^49^ From the micro-array data no conclusions could therefore be drawn about the relative ability of the polymers to stabilise nifedipine in its amorphous state.

For the combinations of MFA with the various excipients a different behaviour to that seen with nifedipine was noted. As composition changed, a fairly clear transition was noted between the opaque, structured and birefringent appearance of the pure MFA systems and the transparent/translucent (or opaque for poly(**d-Ox**-*alt*-GA)) unstructured appearance, without birefringence, of the excipients. This transition is highlighted with a red line on the images of Fig. 3 for a sub-set of the compositions, either side of the transition, for the MFA-deprotected polymer systems (see Fig. S6† for poly(**d-Ox**-*alt*-GA) data). Dp-poly(**d-Ox**-*alt*-PA)–MFA dispersions remained amorphous up to an MFA loading of 38% w/w, showing excellent stability within the experimental timeframe. MFA dispersed in dp-poly(**d-Ox**-*alt*-GA) and poly(**d-Ox**-*alt*-GA) was less stable, with the transition between the amorphous and crystalline phase decreasing from 25 to 14 and 51 to 44 MFA % w/w over the storage time, respectively, with aggregation occurring as before for poly(**d-Ox**-*alt*-GA) ASDs.

### Upscaled ASD formulation

Having established the **d-Ox** copolymers were applicable to ASDs, ASD formulation was upscaled using 20 mg of drug to imitate pharmaceutically relevant conditions, at drug loadings of 50, 33, 25 and 20% w/w, which were achieved by varying the polymer concentration in solution from 5 to 20 mg mL^−1^. Dp-poly(**d-Ox**-*alt*-PA/GA) and drugs were stirred in a 9 : 1 acetonitrile to water solution at 60 °C to ensure full solubility of all components, while poly(**d-Ox**-*alt*-PA/GA) and drugs were stirred in CHCl_3_ at 50 °C. The solvents were then removed, and the samples dried *in vacuo* at 50 °C overnight. As a control, pure drugs were subjected to similar treatment. ASDs were also prepared with HPMC, for comparison with a commercial standard with structural similarities to the **d-Ox** derived polymers (polysaccharide *vs.* synthetic carbohydrate polymer).

The crystallinity and interactions of nifedipine within the polymers were assessed by DSC and FT-IR spectroscopy. Up to the highest drug loading of 50% w/w, all formulations, independent of the polymer, were found to be amorphous, as indicated by the disappearance in the DSC traces of the melting transition at 176 °C of pure nifedipine (Fig. S7†). The observed crystallinity of pure nifedipine contrasts with the 2D inkjet printing results. Here, the greater number of molecules leading to a higher probability of nucleation, as well as the elevated drying temperatures of the bulk ASDs, likely favours crystallisation.^45^

**Fig. 4:**
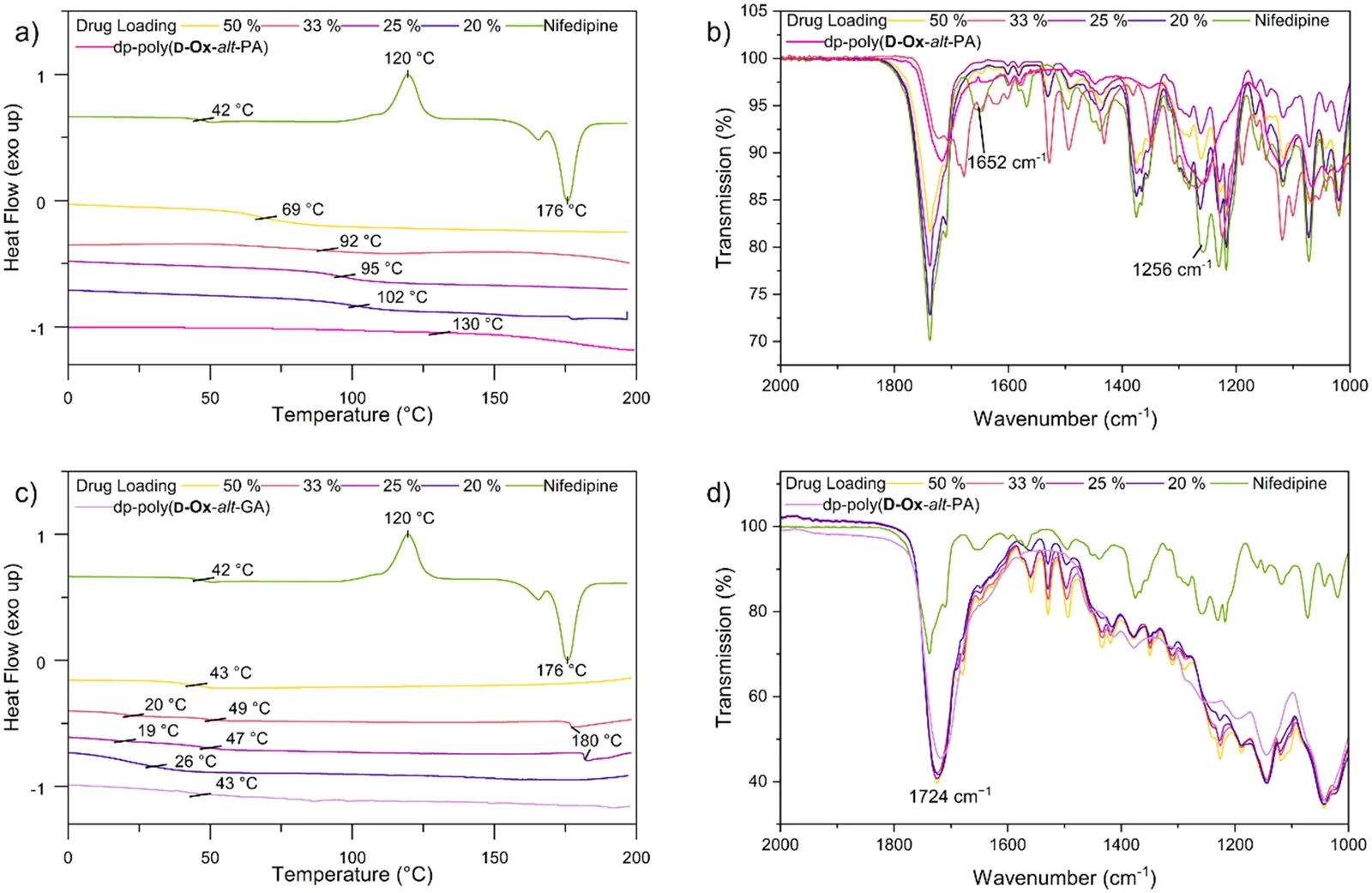
DSC spectra (*exo* up, second cooling cycle), 20 °C min^−1^ of nifedipine ASDs with (a) dp-poly(**d-Ox**-*alt*-PA) (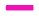) and (c) dp-poly(**d-Ox**-*alt*-GA) (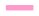). FTIR spectra between 2000 and 1000 cm^−1^ of nifedipine ASDs with (b) dp-poly(**d-Ox**-*alt*-PA) and (d) dp-poly(**d-Ox**-*alt*-GA) at 50% w/w (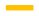), 33% w/w (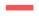), 25% w/w (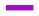), 20% w/w (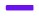).

Dp-poly(**d-Ox**-*alt*-PA) had a clear antiplasticisation effect on nifedipine, increasing the *T*_g_ with increased polymer loading, so that at a drug loading of 20% w/w, the *T*_g_ increased from 42 °C to 102 °C (Fig. 4). Previous studies have found PVP to have the same antiplasticisation effect on nifedipine.^50,51^ Experiments with HPMC and poly(**d-Ox**-*alt*-PA) revealed that both polymers also exerted an antiplasticisation effect on nifedipine (Fig. S7†). As nifedipine is known to crystallise through H-bonding between the carboxylic acid ester and the amine, disrupting these interactions is key to inhibiting crystallinity.^52^ To observe this, previous studies have analysed the changes in the vibrational stretches carbonyl and amine groups.^51,52^ FT-IR spectroscopy revealed dp-poly(**d-Ox**-*alt*-PA) effected a complete disappearance of the *ν*_C=O_ vibration at 1652 cm^−1^ in nifedipine, likely a result of H-bonding between the diol repeat unit and the carboxylic acid ester. This was also observed in HPMC-nifedipine ASDs (Fig. S7†). In contrast, poly(**d-Ox**-*alt*-PA) effected a significant decrease in the *ν*_N=O_ vibration intensity at 1375 cm^−1^ in nifedipine.

**Fig. 5:**
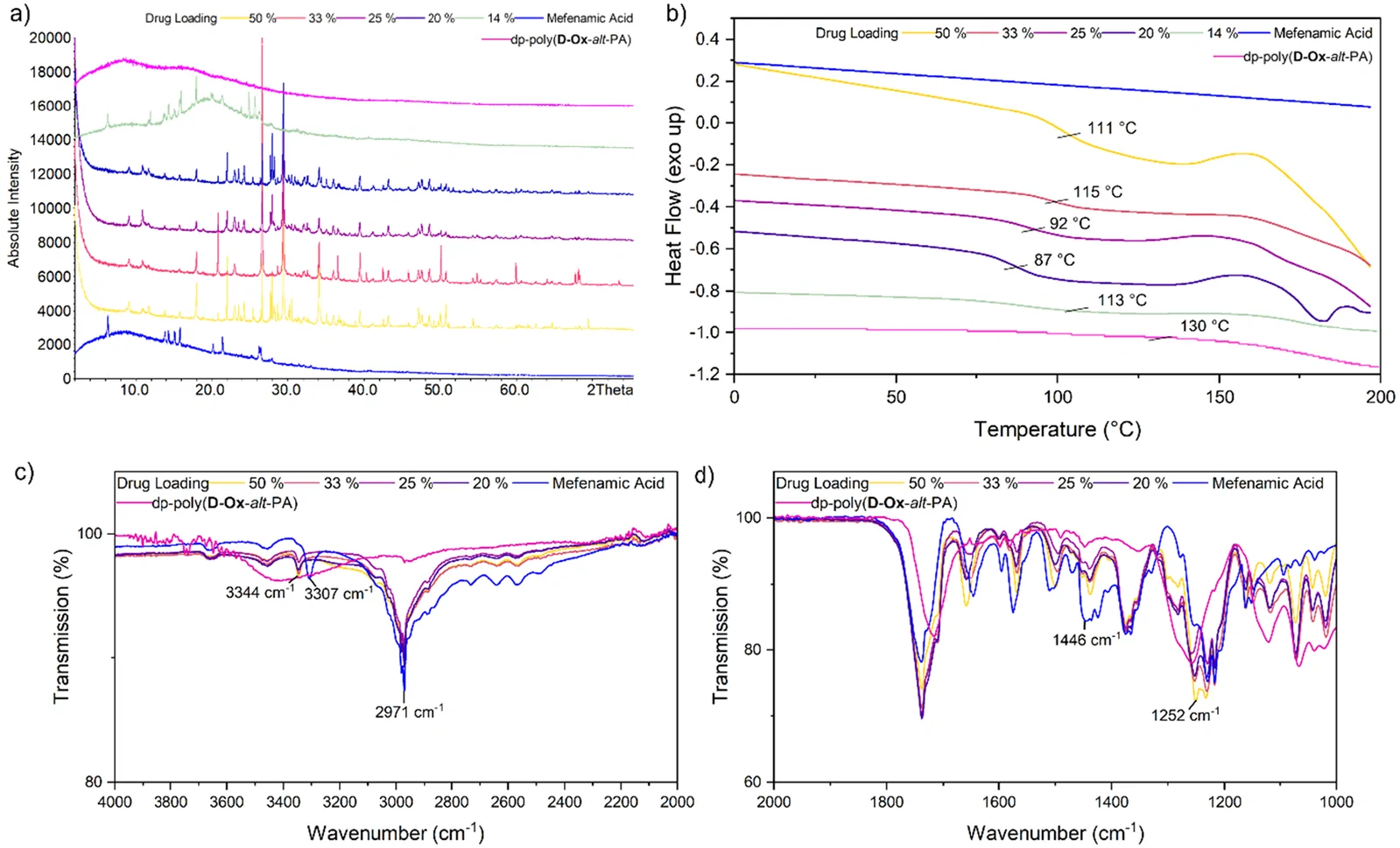
dp-Poly(**d-Ox**-*alt*-PA) (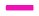) MFA (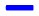) ASDs at 50% w/w (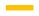), 33% w/w (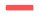), 25% w/w (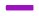), 20% w/w (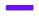), 14% w/w (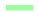). (a) Stacked PXRD patterns of dp-poly(**d-Ox**-alt-PA)–MFA ASDs with MFA and dp-poly(**d-Ox**-*alt*-PA). (b) Stacked DSC traces (*exo* up), 20 °C min^−1^. (c) FTIR spectra between 4000 and 2000 cm^−1^. (d) FTIR spectra between 2000 and 1000 cm^−1^.

Analyses of MFA formulations were next performed. DSC proved inefficient at assessing their crystallinity, due to the sublimation of MFA occurring before melting (∼210 °C and 233 °C respectively).^53,54^ Powder X-Ray Diffraction (PXRD) analysis was therefore carried out to evaluate the crystallinity of polymer dispersions, including with HMPC (Fig. S8†). For all formulations, PXRD analysis showed crystalline regions at drug loadings between 20–50% w/w. Notably, new crystalline patterns were observed compared to the pure MFA PXRD pattern (form I), at all drug loadings, indicating polymorphic MFA crystals in the formulations (forms I and II).^55^ At MFA loading of 14% w/w, a halo pattern became visible for dp-poly(**d-Ox**-*alt*-PA) ASDs, indicating an amorphous nature (Fig. 5a). This was in line with the microarray data, which predicted that dp-poly(**d-Ox**-*alt*-PA) dispersions would be most effective in stabilising amorphous MFA.

Like nifedipine, MFA crystallises through H-bonding between the amine and carboxylate or carboxylate and carboxylate groups.^56^ Previous FT-IR spectroscopic studies have shown that whilst stabilising amorphous MFA, HPMCAS causes a complete disappearance of its *ν*_(N–H)_ vibration at 3304 cm^−1^.^57^ Here, dp-poly(**d-Ox**-*alt*-PA) induced a significant shift of the MFA *ν*_(N–H)_ vibration shift from 3304 to 3346 cm^−1^, indicating a shortening of the N–H bond (Fig. 5c and d). The transition from form I MFA to form II, a more soluble polymorph, is known to be accompanied by a shift in the FTIR spectrum from 3313 to 3347 cm^−1^.^58^ The FT-IR data, combined with the additional crystalline PXRD patterns, thus indicates that dp-poly(**d-Ox**-*alt*-PA) assists the polymorphic crystallisation of MFA from form I to form II.

DSC analysis of dp-poly(**d-Ox**-*alt*-PA) dispersions revealed a single *T*_g_, indicating of a single amorphous phase with no phase separation. At high drug loadings (20–50% w/w), the overall *T*_g_ of the dispersion decreased with decreasing drug loading (increasing polymer loading), suggesting the polymer is not exerting an antiplasticisation effect, in contrast to what was observed with nifedipine. However, below 20% w/w drug loading, an antiplasticisation effect was observed, raising the *T*_g_ of the ASD back up to 113 °C (Fig. 5b). In agreement with PXRD analysis, at MFA loading of 14% w/w, the disappearance of an exothermic event beginning at 160 °C, previously attributed to traces of MFA crystallinity,^57^ was noted compared to higher drug loadings. In comparison, HPMC–MFA ASDs showed no further improvement in amorphicity when decreasing the MFA loading below 20% w/w (Fig. S8†). Overall, DSC and PXRD analyses converge towards a single ASD of dp-poly(**d-Ox**-*alt*-PA) and MFA, with some crystalline drug separating out.

PXRD analysis of dp-poly(**d-Ox**-*alt*-GA) dispersions qualitatively showed a less significant reduction in MFA crystallinity compared to dp-poly(**d-Ox**-*alt*-PA) (Fig. S8†). Notably, like for dp-poly(**d-Ox**-*alt*-PA) ASDs, the PXRD pattern of dp-poly(**d-Ox**-*alt*-GA)–MFA dispersions displayed evidence of polymorphic MFA crystals formation. Finally, despite similar *T*_g_s, dp-poly(**d-Ox**-*alt*-GA) was found to exert a significant plasticisation effect on MFA, lowering its *T*_g_ from 42 °C to 1 °C and 14 °C at 14 and 50% w/w MFA loadings, respectively, as observed by DSC (Fig. S8†).

Overall, the results from the micro-array microscopy screening were in fair agreement with the PXRD and DSC data, given the differences in sample size (mg *vs.* ng) and range of composition space investigated in the bulk and micro-array approaches.

### Dissolution studies

The solubility of nifedipine and MFA in DI water when formulated as ASDs was next assessed and compared to the solubility of the free drugs. Nifedipine ASD dissolution studies were carried out with ASDs of 50% w/w as DSC analysis suggested that at 50% w/w, nifedipine remained amorphous in all polymers. MFA ASD dissolution studies were carried out with ASDs of 14% w/w as microarray data and PXRD analysis suggested that at 14% w/w, MFA is partially amorphous in dp-poly(**d-Ox**-*alt*-PA) and HPMC.

**Fig. 6:**
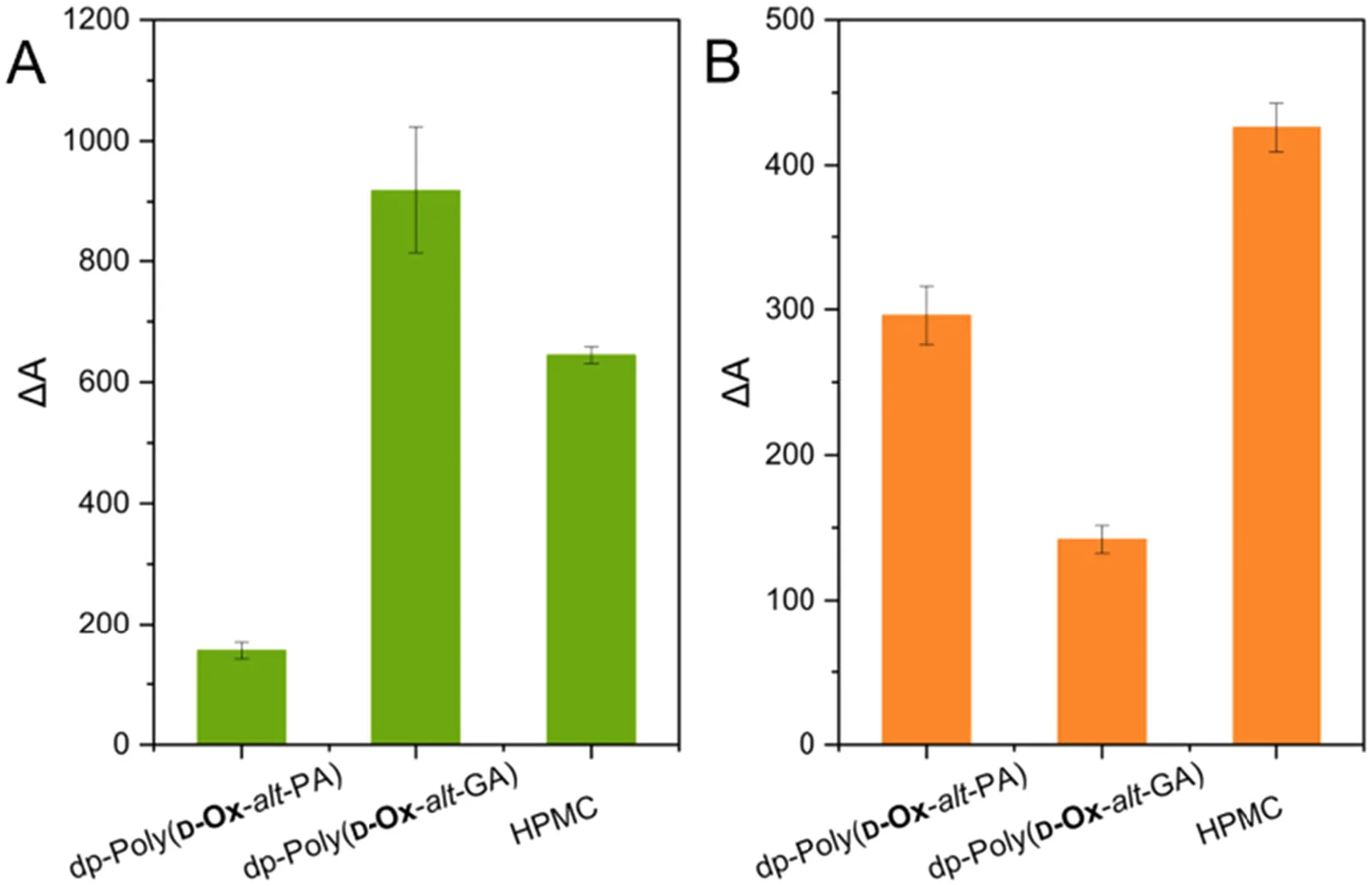
(A) relative UV absorbance at 340 nm in DI water after 2 hours when dispersing nifedipine in polymer *versus* the free drug (triplicate measurement). (B) Relative UV absorbance at 292 nm in DI water after 2 hours when dispersing MFA in polymer *versus* the free drug (triplicate measurement).

Both nifedipine and MFA showed improved apparent solubility in water when formulated with polymers compared to the drugs alone (as shown by relative differences in UV-Vis absorbance, Δ*A*% values; Fig. 6).

Dispersions of nifedipine within dp-poly(**d-Ox**-*alt*-GA) was shown to improve its solubility in water after 2 hours by 918%, compared to increase in solubility by 155% for dp-poly(**d-Ox**-*alt*-PA) and 645% for HPMC. As predicted by the microarray and bulk results, dp-poly(**d-Ox**-*alt*-PA) enhanced the solubility of MFA most effectively, increasing the solubility of MFA by 296%, although Δ*A*% values were lower than with HPMC, which increased solubility by 426%. As expected, higher solubility enhancement of nifedipine was observed with dp-poly(**d-Ox**-*alt*-PA/GA), compared to poly(**d-Ox**-*alt*-PA/GA); 155 *vs.* 45% and 918 *vs.* 36%, respectively (Fig. S12†). This was expected as the deprotected polymers were water soluble, and the solvation of nifedipine would likely be aided by the solvation of these polymers.

From the data, the structure of the polymer is key for the efficacy of the ASDs to increase the drug apparent solubility in water. The superior ability of dp-poly(**d-Ox**-*alt*-GA) to solubilise nifedipine may be attributed to their similar *T*_g_s (42–43 °C), compared to HPMC (*T*_g_ = 143–147 °C) or dp-poly(**d-Ox**-*alt*-PA) (*T*_g_ = 130 °C). As such, it meets the criteria of a polymer with a *T*_g_ 0–50 °C higher than the drug.^15^ The superior ability of dp-poly(**d-Ox**-*alt*-PA) to solubilise MFA may be attributed, as shown by FT-IR spectroscopy, to the ability of dp-poly(**d-Ox**-*alt*-PA) to disrupt key N–H bonding interactions required for MFA crystallisation. Additionally, PXRD analysis suggests that dp-poly(**d-Ox**-*alt*-PA) facilitates the formation of polymorphic MFA which may add to the improved solubility.^58^

The poor ability of dp-poly(**d-Ox**-*alt*-GA) to improve MFA apparent solubility in water can be rationalised by the low *T*_g_ of the 14% w/w dispersion (1 °C). Such plasticisation effect would allow for molecular mobility and crystallisation. This result was correctly predicted by 2D inkjet-printing which showed that MFA remained dispersed within dp-poly(**d-Ox**-*alt*-PA) up to 38% w/w after 2 months, whereas it would begin to crystallise out of dp-poly(**d-Ox**-*alt*-GA) at 25% w/w after two days.

### Computational modelling of solubility parameters

Although matching drugs and polymers based on their *T*_g_ proved effective to obtain dispersions that enhance the solubility of nifedipine in water, this was not the case for MFA dispersions. The drug and polymer miscibility with each other were thus also evaluated using molecular dynamics, by calculating their solubility parameters (see ESI, Table S2†). Materials with similar solubility parameters are expected to show enhanced mixing, so that the closer the solubility parameter values of the drug and of the polymer, the more physically stable the resulting ASD would be.^59,60^

Dp-poly(**d-Ox**-*alt*-PA) was found be more polar than dp-poly(**d-Ox**-*alt*-GA), with solubility parameters of 19.0 and 11.7 (MJ m^−3^)^½^ respectively. MFA, with a solubility parameter of 18.5 (MJ m^−3^)^½^ is therefore most compatible with dp-poly(**d-Ox**-*alt*-PA), according to this model. These computational results may shed light on the improved performance of dp-poly(**d-Ox**-*alt*-PA) in stabilising amorphous MFA compared to dp-poly(**d-Ox**-*alt*-GA), governed by well-matched solubility rather than well-matched *T*_g_. It should be noted that nifedipine (22.1 (MJ m^−3^)^½^) and dp-poly(**d-Ox**-*alt*-GA) do not fit this model, with a difference in solubility parameters of 9.8 (MJ m^−3^)^½^ despite the high performance of dp-poly(**d-Ox**-*alt*-GA)-nifedipine ASDs. These results highlight the complexity in predicting the efficiency of ASDs, especially with new polymers where limited data is available.

## Conclusions

The controlled copolymerisation of a xylose-derived oxetane into fully biobased polyesters allowed facile tuning of the polymer properties to complement the properties of two poorly water-soluble drugs, nifedipine and MFA, which is nontrivial through conventional methods such as polymer side-chain modification. Using high-throughput 2D inkjet printing and optical microscopy, the polymers were found to maintain MFA in an amorphous state at 38% w/w in dp-poly(**d-Ox**-*alt*-PA) and 25% in dp-poly(**d-Ox**-*alt*-GA). Microarray data showed a good correlation with the performance of the polymers in bulk and their effectiveness at improving the drug water solubility. Analysis of bulk dispersions revealed the nature of the polymer–drug interactions varied significantly with the polymer structure. FT-IR spectroscopy indicated that the acetal-deprotected polymers were effective in disrupting key H-bonding in both nifedipine and MFA. Drug dissolution studies showed that dp-poly(**d-Ox**-*alt*-GA) improved the solubility of nifedipine by 918%, a significant improvement compared to commercial standard HPMC, which improved the solubility of nifedipine by 645%. This was attributed to the low *T*_g_ of dp-poly(**d-Ox**-*alt*-GA) being more compatible with nifedipine (both 42–43 °C). MFA dissolution studies showed that the solubility enhancement effected by dp-poly(**d-Ox**-*alt*-PA) compared well with that of HPMC (296% *vs.* 426%, respectively). These proof-of-concept results highlight the potential of bioderived (specifically xylose-derived) polyesters made by ROCOP as a promising platform for ASDs, with several tuneable features (*e.g.*, molar mass and dispersity, sequence, architecture) yet to be explored.

## Author contributions

The manuscript was written through contributions of all authors. All authors have given approval to the final version of the manuscript. EC: all experimental work and preparation of the manuscript. AH: control of the piezo electric inkjet printer (Sciflexarray S5, Scienion). SMF: initial experiments which became the foundation of this work. JFM: computational modelling and manuscript review and editing. JCB: conceptualisation of inkjet printing, manuscript review and editing VT: conceptualisation, manuscript writing, review and editing, supervision. AB: conceptualisation, manuscript writing – review and editing, supervision and funding acquisition.

## Conflicts of interest

There are no conflicts to declare.

## Supplementary Material

LP-002-D4LP00203B-s001

## Data Availability

The data supporting this article have been included as part of the ESI.†
