# Supplementary material for: d-Xylose oxetane copolymers as bioderived and tuneable polyesters for amorphous solid dispersions
Source: RSC Appl Polym. 2024 Sep 4;2(6):1104–12. doi: 10.1039/d4lp00203b (PMC13591373; doi:10.1039/d4lp00203b)
Supplement: Supplementary file 1 [file LP-002-D4LP00203B-s001.pdf]

Supplementary Information (SI) for RSC Applied Polymers.
This journal is © The Royal Society of Chemistry 2024

Supporting Information

D-Xylose oxetane copolymers as bioderived and tuneable polyesters for amorphous solid dispersions

Ella F. Clark,[a] Alexandra Howard,[b] Sebastian D. Morales Feliu,[a] James F. McCabe,[c] Jonathan C. Burley,[b] Vincenzo Taresco[d,] and Antoine Buchard[e*]*

[a] Department of Chemistry, University of Bath, Bath, BA2 7AY, UK.

[b] University of Nottingham, School of Pharmacy, Nottingham, NG7 2RD, UK.

[c] Early Product Development and Manufacturing, Pharmaceutical Sciences, R&D AstraZeneca, Macclesfield, SK10 2NA, UK.

[d] University of Nottingham, School of Pharmacy, Nottingham, NG7 2RD, UK.

[e] Department of Chemistry, University of York, York, YO10 5DD, UK.

Emails: antoine.buchard@york.ac.uk
vincenzo.taresco@notthingham.ac.uk

Table of Contents

1. Materials and Methods S3
2. Catalyst Screening for the ROCOP of **D-Ox** and PA S7
3. Polymers Characterisation S8
   3.1 dp-poly(**D-Ox**-*alt*-PA) data S8
   3.2 dp-poly(**D-Ox**-*alt*-GA) data S9
4. Amorphous Solid Dispersions Characterisation S10
   4.1 Microscopy images of the poly(**D-Ox**-*alt*-GA) MFA microarray S10
   4.2 DSC traces and FTIR spectra of Nifedipine ASDs S11
   4.3 DSC traces, PXRD patterns and FTIR spectra of MFA ASDs S12
   4.4 UV-vis spectra S14
   4.5 Computational Modelling S15
   4.6 Summary table S16

1. Materials and Methods

Mefenamic Acid and hydroxypropylmethyl cellulose (M_n ≈ 86,000 g/mol, T_g = 165 °C) were purchased from Merck. Nifedipine and trifluoracetic acid were purchased from ThermoFisher Scientific. All solvents were purchased from Merck. Glutaric anhydride was triply recrystallized from anhydrous toluene prior to use. Phthalic anhydride was stirred over hot toluene overnight, filtered, then recrystallized from anhydrous toluene twice prior to use. Slides for microarray printing were purchased from Corning with 75x 25 mm dimensions, thickness = 0.96 to 1.06 mm. The slides were pretreated with oxygen plasma for 10 mins at 100 % and then added to 480ml of toluene and 20mL of 3-(trimethoxysilyl) propyl acrylate. They were then heated to 50˚C for 24 hours under argon gas and dried *in vacuo* for 12 hours. All other reagents were used as received. **D-Ox** was synthesized according to the literature procedure.[32]

^{1}H NMR spectra were recorded on a Bruker 500 MHz instrument and referenced to residual solvent peaks. Coupling constants are given in Hertz. Polymer conversions and percentage deprotection were determined by relative integration of suitable signals in the ^{1}H NMR spectra.

THF Size-exclusion chromatography (SEC) was carried out using a THF eluent on an Agilent 1260 Infinity series instrument, equipped with two PLgel 5 μm MIXED−D 300 × 7.5 mm columns in series. Polymer samples were dissolved at a concentration of 1 mg mL^{-1} in THF and eluted at 1 mL min^{-1} at 35 °C. Samples were detected with a differential refractive index (RI) detector and data processed using Agilent GPC/SEC software. Number-average molar mass ($M_{n,SEC}$), and dispersities, ($Đ_M$ (M_w/M_n)) were analyzed calculated against a polystyrene calibration (11 polystyrene standards of narrow molar mass, ranging from M_w 615 - 568000 Da).

DMAc Size-exclusion Chromatography **(SEC)** was carried out using a N,N-Dimethylacetamide (DMAc) with LiBr (0.1 w/w %) eluent on a Shimadzu High Performance Liquid Chromatograph i-Series LC-2050C LT instrument, equipped with two Polargel-M 300 x 7.5 mm columns in series. Polymer samples were dissolved at a concentration of 1 mg mL^{-1} and eluted at 1 mL min^{-1} at 50 °C. Samples were detected with a differential refractive index (RI) detector and data processed using the Shimadzu LabSolutions software. Number average molar mass and dispersities, were calculated against a poly(ethylene glycol)/poly(ethylene oxide) calibration (11 PEG standards of narrow molar mass, ranging from 194 - 1378000 Da).

Powder X-Ray diffraction (PXRD) experiments were conducted on samples loaded on films with a STOE STADI P double setup in transmission and reflection mode (Cu radiation, monochromated. radiation = 1.54060 Cu generator: 40 kV, 40 mA, 2Theta (begin, end, step) = 2.000, 75,365, 0.015 600.0 sec/step, total acquisition time = 20 minutes.

Differential scanning calorimetry (DSC) was carried out using a MicroSC multicell calorimeter from Setaram. The measurement cell and the reference cell were both a 1 mL Hastelloy C cell; a mass of 3-6 mg of polymeric material was loaded into the measurement cell with the reference cell empty.

The experiment was performed under nitrogen gas and the sample heated and cooled at a rate of 20 K min^{-1}. A second heating and cooling cycle was carried out immediately following completion of the first. Glass transition temperatures (T_gs) are reported from the second heating cycle unless otherwise stated. The Calisto program was employed to collect and process the data. Data was plotted using Origin 2023.

FT-IR analysis was done using a PerkinElmer Inc. Spectrum 100 FT-IR Spectrometer. Universal ATR enabling wavelengths from 650-4000 cm^{-1} (15 µm to 2.5 µm).

General Procedure for Polymer Synthesis. Following an adapted literature procedure;[36, 39] under an argon atmosphere, Al Trisphenolate (25.6 mg, 5 x 10^{-2} mmol, 1 equiv.), PPNCl (28.7 mg, 5 x 10^{-2} mmol, 1 equiv.) and the cyclic anhydride (10 mmol, 200 equiv.) were added to a 2.35 mol L^{-1} σ-dichlorobenzene solution of oxetane (1.72 g, 10 mmol, 200 equiv.). The vessel was heated to 100 °C. The reaction progress was monitored by ^{1}H NMR spectroscopy spectroscopy by relative integration of anomeric protons in **D-Ox** at 6.26 and 5.95 ppm of the monomer and polymer respectively. The reaction was terminated by cooling the reaction vessel down. The resultant sticky solid was dissolved in $CHCl_3$ (2 mL) and the polymer was precipitated with cold Et_2O (10 mL). The suspension was then centrifuged (2900 rpm, 5 minutes) and the precipitate was collected. The solid phase was redissolved in $CHCl_3$ (2 mL) and reprecipitated twice more from cold Et_2O before being dried *in vacuo* at 100 °C to yield the polyester.

General Procedure for Acid-catalyzed Polymer Deprotection. Following an adapted literature procedure;[35] poly(**D-Ox**-*alt*-PA/GA) (1 g) with dissolved in DCM (8 mL) and cooled to 0 °C. A 4:1 TFA:H_2O solution (36 mL) was added and stirred for 8 h with aliquots taken every hour. The product was precipitated from cold Et_2O, and the resulting suspension was centrifuged (3500rpm, 5 min). The solid phase was collected and rinsed with cold Et_2O until the supernatant was neutral by a litmus test. The solid phase was collected, but not dried, to yield poly(**D-Ox**-*alt*-PA) and poly(**D-Ox**-*alt*-GA) deprotected at 97 % and 93 % (calculated by relative integration of protected and deprotected anomeric environments of the ^{1}H NMR spectra, in DMSO-d_6). The product was left in Et_2O at −4 °C to prevent the polymer becoming dry and insoluble.

Microarray Printing. Polymer and drug solutions in DMSO (1 mg mL^{-1} and 5 mg mL^{-1} respectively) were deposited on glass microscope slides, with a piezo electric inkjet printer (Sciflexarray S5, Scienion) using a 90µm orifice nozzle. The droplet size was controlled by the values of the electrical pulse and voltage. To verify droplet size, DMSO solution droplets with nominal volumes ranging from 250 to 280 pL were dispensed at a 3 kHz jetting frequency by adjusting the voltage and pulse between 98 and 105 V and 45−55µs, respectively. Two spots at each weight/weight % ratio of drug/polymer were printed, varying the order deposition order (printing the drug 1st and the polymer 2nd to form the first spot and the reverse order for the second spot). The nozzle was washed with DMSO in between each printing cycle, as part of the automated printing−washing loop. Printed DMSO solutions were left to evaporate for two days in the printer cage at around 29.8 °C and 55 % of Relative Humidity and

subsequently stored in a desiccator avoiding moisture contamination. Previous reports have found that DMSO normally evaporates in a time frame of 100−120 min for the biggest printed droplets (around 65−70 pl).[40] The microarrays were investigated two days after printing using an Advanced Polarizing Microscope (HS1microscope), Prior LuxPOLTM with 12 V, and a 30 W halogen lamp with variable brightness control to analyze the crystallinity of the drugs in these amounts.

Microscopy images were also collected for the micro-array samples after storage at room temperature for approximately two months. Phase contrast and cross-polarised images were collected using a Nikon NiE automated microscope equipped with a 4x lens at room temperature operating using transmitted light. Similar acquisition parameters were used for all images, except for the polarised images which required a longer exposure than the phase contrast. Imaging was performed such that the drug-first printed spots appear on the left and the excipient-first on the right replace.

Dp-poly(D-Ox-*alt*-PA)-drug dispersions. Nifedipine or Mefenamic Acid (20 mg, 58 and 83 μmol respectively) was added to a solution of dp-poly(**D-Ox**-*alt*-PA/GA) in a 9:1 Acetonitrile to water solution (5 − 20 g L^{-3}) and stirred overnight at 60 °C. The solvent was then removed using the DrySyn Spiral Evaporator at 50 °C and dried in vacuo at 50 °C.

Poly(D-Ox-*alt*-PA)-drug dispersions. Nifedipine or Mefenamic Acid (20 mg, 58 and 83 μmol respectively) was added to a solution of poly(**D-Ox**-*alt*-PA/GA) in chloroform (5 − 20 g L^{-3}) and stirred overnight at 50 °C. The solvent was then removed using the DrySyn Spiral Evaporator at 50 °C and dried in vacuo at 50 °C overnight.

Dissolution Analysis. The ASDs or free drugs were stirred in DI water, at a drug concentration of 5 mg mL^{-1} for two hours at room temperature (10 mg of ASD in 1 mL of DI water at a Nifedipine loading of 50 %w/w ; 35.7 mg of ASD in 1 mL of DI water at a MFA loading of 14 % w/w). HPMC−MFA samples were tested at a MFA concentration of 2.5 mg mL^{-1} to prevent gelling (17.5 mg of ASD in 1 mL of DI water at a MFA loading of 14 % w/w). This was done in triplicate. Insoluble polymer or drug was removed by filtration through a PTFE filter and a UV-vis spectra of the filtrate was recorded between 200 and 800 nm. Any overlapping absorbance from the polymer was negated by using a polymer solution as the baseline, at a polymer concentration to match the polymer concentration of the dissolution studies (5 g L^{-1} for Nifedipine and 30.7 g L^{-1} for MFA).

The absorbance values of the ASDs (A_{ASD}) were normalized against the absorbance of the free drug in water (A_D) at the same wavelength (340 and 290 nm for nifedipine and MFA respectively) (**Equation 1**, at a polymer concentration to match the polymer concentration of the dissolution studies (5 g L^{-1} for Nifedipine and 30.7 g L^{-1} for MFA).

The solubility of these drugs could then be compared using this value (ΔA %).

$$\Delta A\ \% = \left(\frac{A_{ASD} - A_D}{A_D}\right) \times 100 \quad \textbf{Equation 1}$$

Molecular Dynamics Simulations. All energy minimisations and MD simulations were performed using Materials Studio (Biovia version 2022). Polymers consisting of 20 monomer units were built using the polymer builder with Materials Studio and geometry optimised using the COMPASS III forcefield. Amorphous cells were then constructed consisting of 20 molecules of the '20mer' polymer. Using the Forcite module, the amorphous cells were equilibrated at 298K using a NVT dynamics simulation (5ps in length, velocity scale thermostat) followed by a longer NPT run ay 298K (500ps, velocity scale thermostat, Berendsen barostat). Finally, a cohesive energy density calculation (CED) was performed to obtain the solubility parameter (defined as the square root of the CED) for the amorphous systems. A similar process was used for amorphous cells of nifedipine and mefenamic acid.

2. Catalyst Screening for the ROCOP of D-Ox and PA

Table S1. Catalyst Screening for the ROCOP of **D-Ox** and PA.

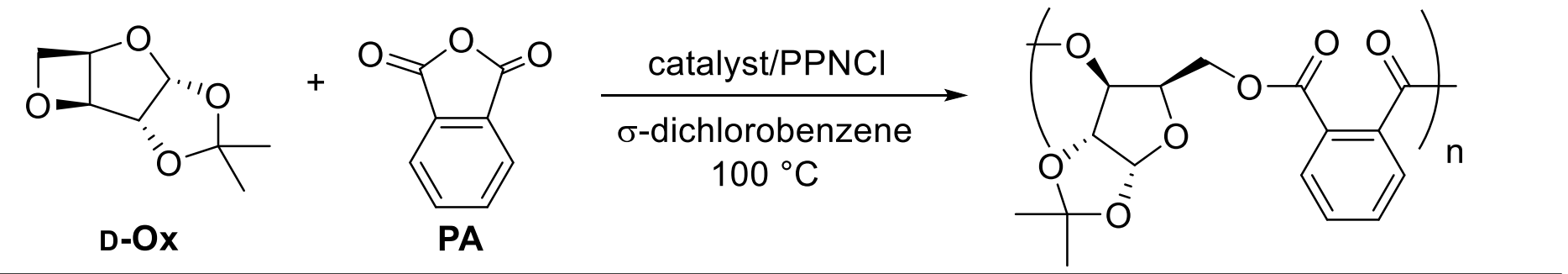

catalyst/initiator systems tested:

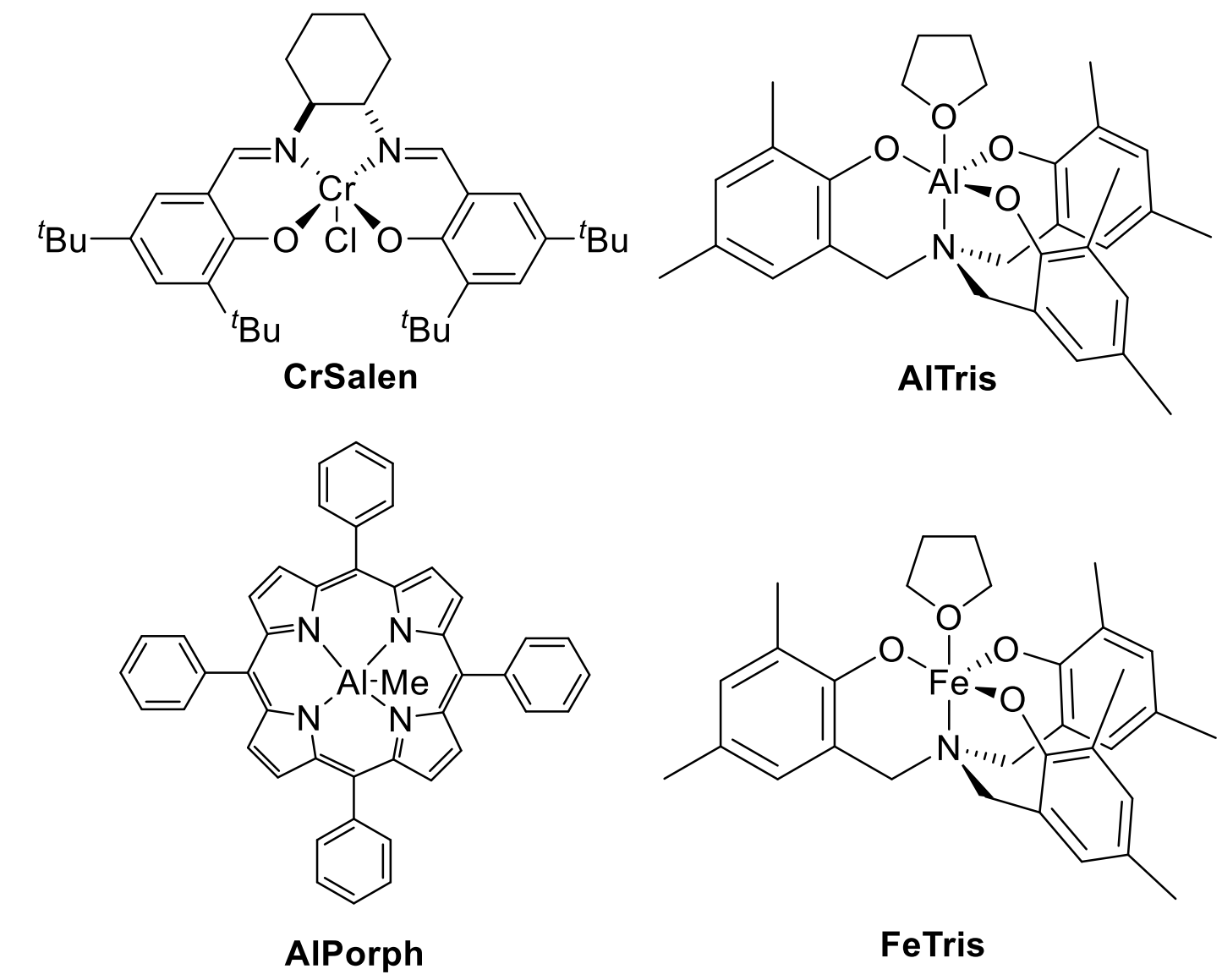

Entry[a]	Catalyst	Temp (°C)	Time (h)	Conv. %.[b]	Select %[c]	TOF[d]	$M_{n,SEC}$ ($Đ_M$)[e]	Entry[a]
1	**CrSalen**	100	24	62	>99	5.2	3,500 (1.21)	1
2	**CrSalen**	100	50	80	>99	3.2	7,500 (1.28)	2
3	**AlTris**	100	24	78	>99	6.5	16,400 (1.37)	3
4	**AlTris**	100	48	>99	>99	-	18,000 (1.86)	4
5	**AlPorph**	100	20	90	77	9.0	9,000 (1.42)	5
6	**FeTris**	100	24	35	>99	2.9	6,500 (1.30)	6

[a] Reactions carried out at [**D-Ox**]$_0$= 1.34 mol L^{-1} in *σ*-dichlorobenzene with PPNCl at a [**D-Ox**]$_0$[PA]$_0$[cat]$_0$[PPNCl]$_0$ ratio of 200:200:1:1. [b]Conversion of **D-Ox** determined by ^{1}H NMR spectroscopy by relative integration of anomeric protons in **D-Ox** ($CDCl_3$, δ= 6.26 ppm (d,J= 3.7 Hz)) and poly(**D-Ox**-*alt*-PA) ($CDCl_3$, δ = 5.95 ppm (d, J = 3.6 Hz)). [c]Selectivity of ester vs ether links determined by ^{1}H NMR spectroscopy using the relative integration of anomeric ester environments ($CDCl_3$, δ= 4.55 ppm (^{1}H, d, J= 3.6 Hz)) *vs* methylene ether environments ($CDCl_3$, δ = 3.57 (2H, m)). [d]TOF = (moles of **D-Ox** consumed)×(moles of catalyst)$^{-1}$×(time of reaction)$^{-1}$. [e]M_n in g mol^{-1}, calculated by SEC relative to polystyrene standards in tetrahydrofuran (THF) eluent; $Đ_M = M_w/M_n$.

3. Polymers Characterisation

3.1 dp-poly(D-Ox-*alt*-PA) data

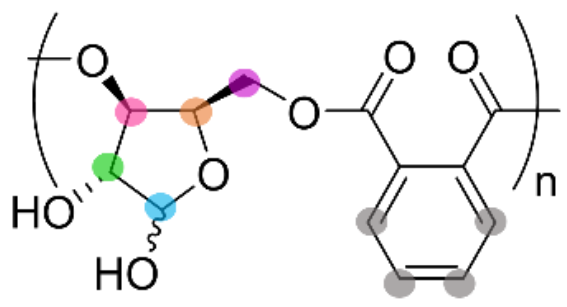

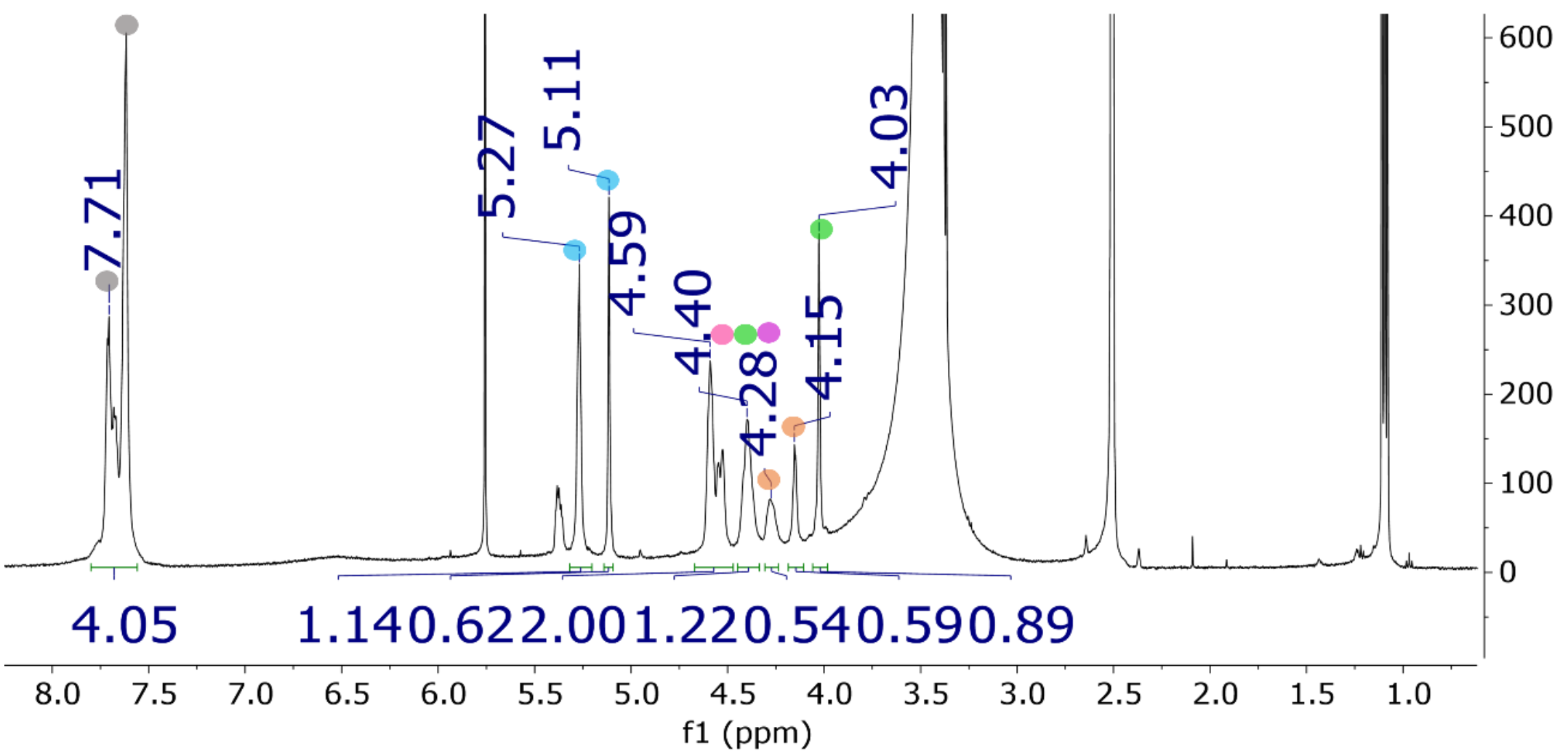

Figure S1. ^{1}H NMR (DMSO-d_6) of >99 % deprotected dp-poly(**D-Ox**-*alt*-PA) (DCM residual signal at 5.75 ppm, water residual signal at 3.33, DMSO residual signal at 2.50 ppm and Et_2O residual signal at 3.38 and 1.09 ppm).

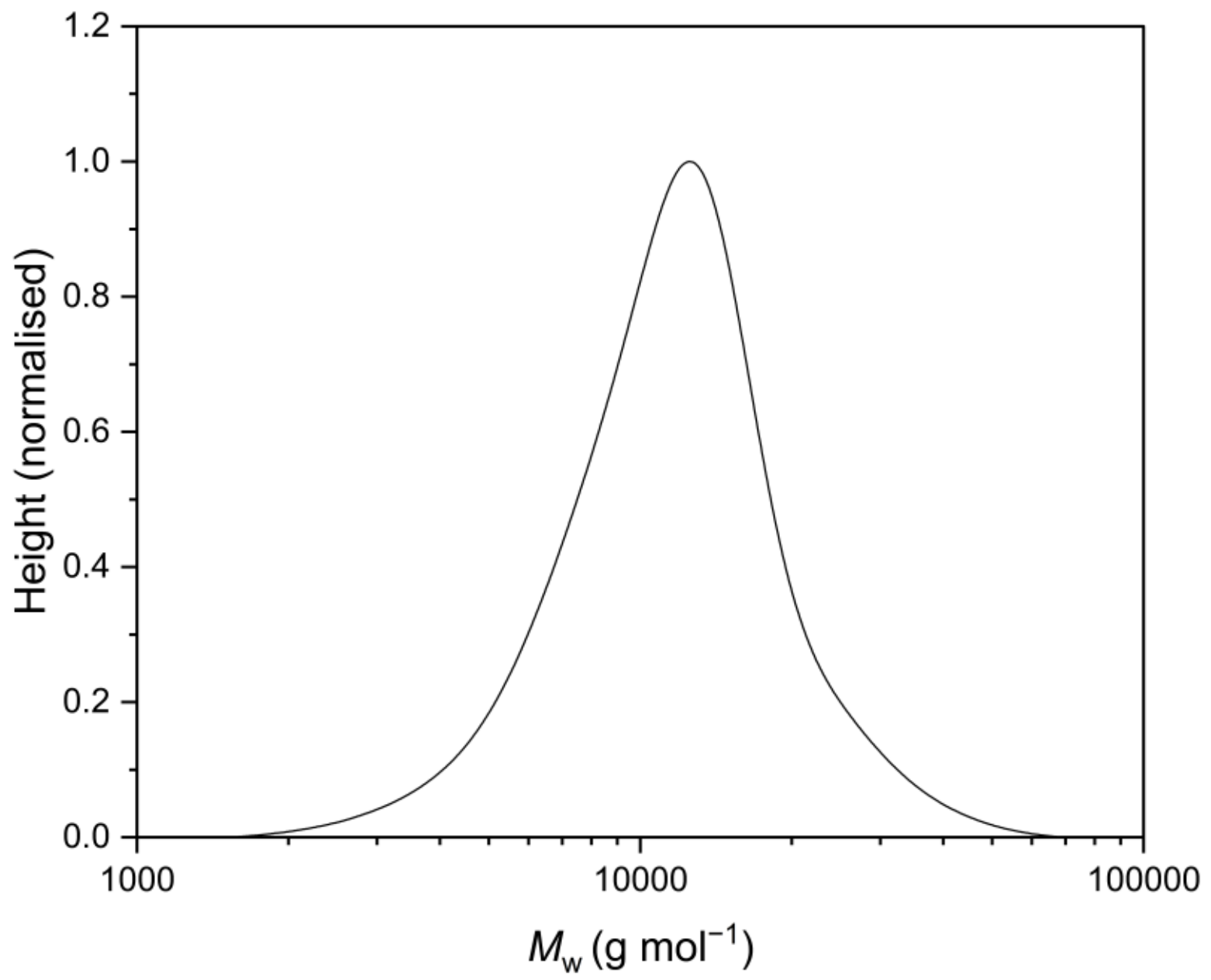

Figure S2. SEC trace for dp-poly(**D-Ox**-*alt*-PA)($M_{n,SEC}$= 10,100 g mol^{-1}, $Đ_M$ = 1.29).

3.2 dp-poly(D-Ox-*alt*-GA) data

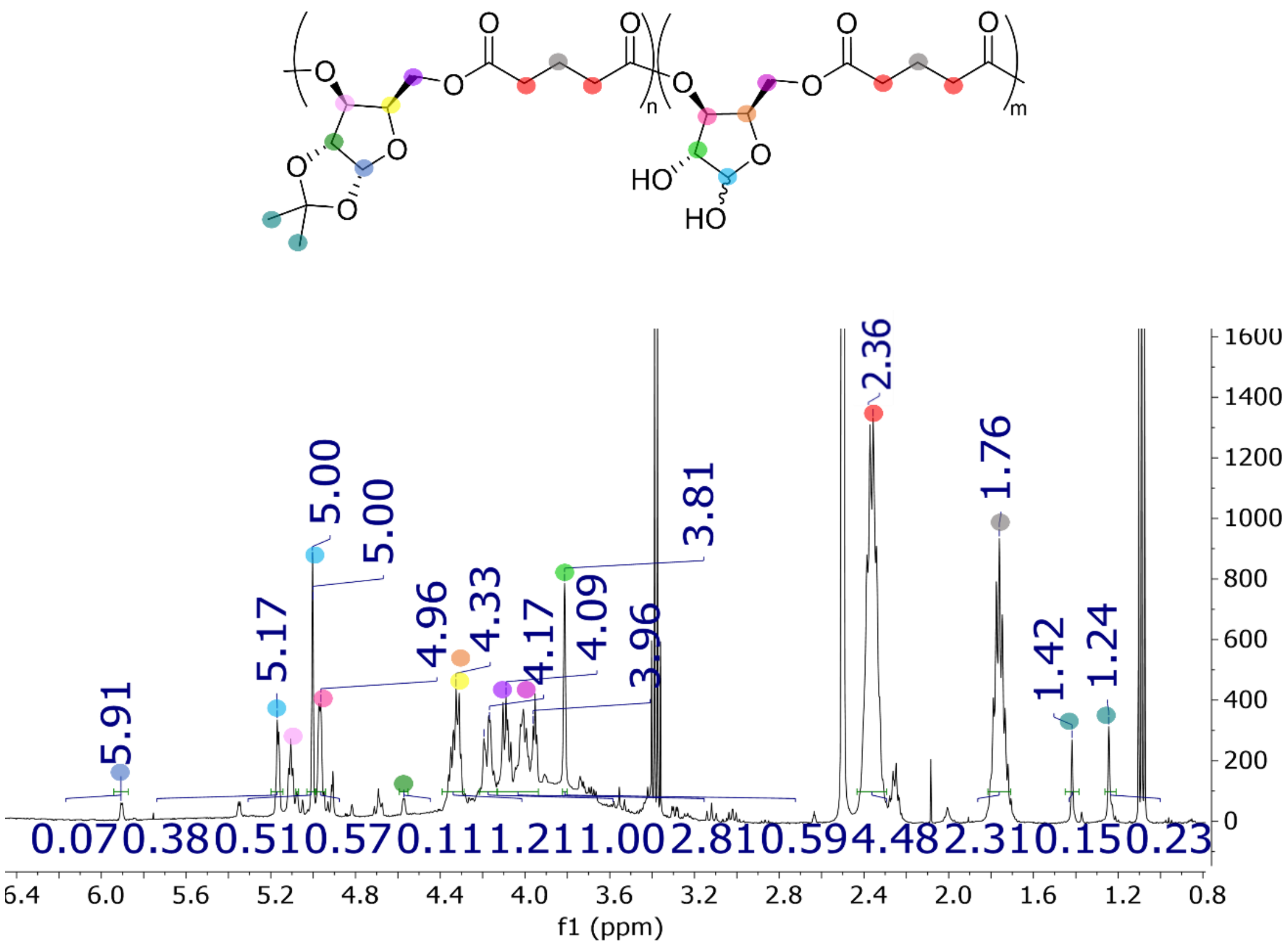

Figure S3. ^{1}H NMR (DMSO-d_6) of 93% deprotected dp-poly(**D-Ox**-*alt*-GA) (Et_2O residual signal at 3.38 and 1.09 ppm, DMSO residual signal at 2.50 ppm).

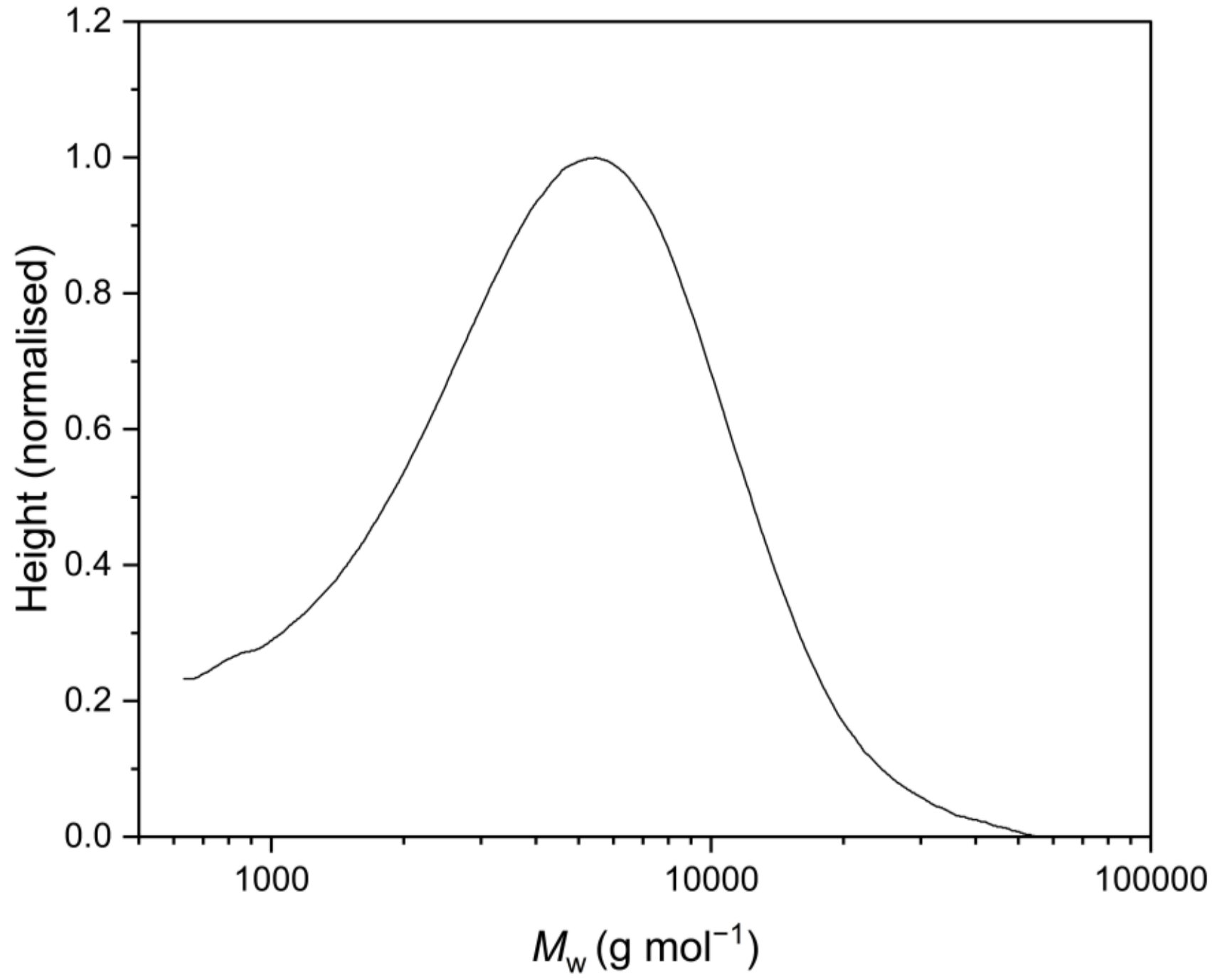

Figure S4. SEC trace for dp-poly(**D-Ox**-*alt*-GA)($M_{n,SEC}$= 3,000 g mol^{-1}, $Đ_M$ = 2.18).

4. Amorphous Solid Dispersions Characterisation

4.1 Microscopy images of the poly(D-Ox-alt-GA) MFA microarray

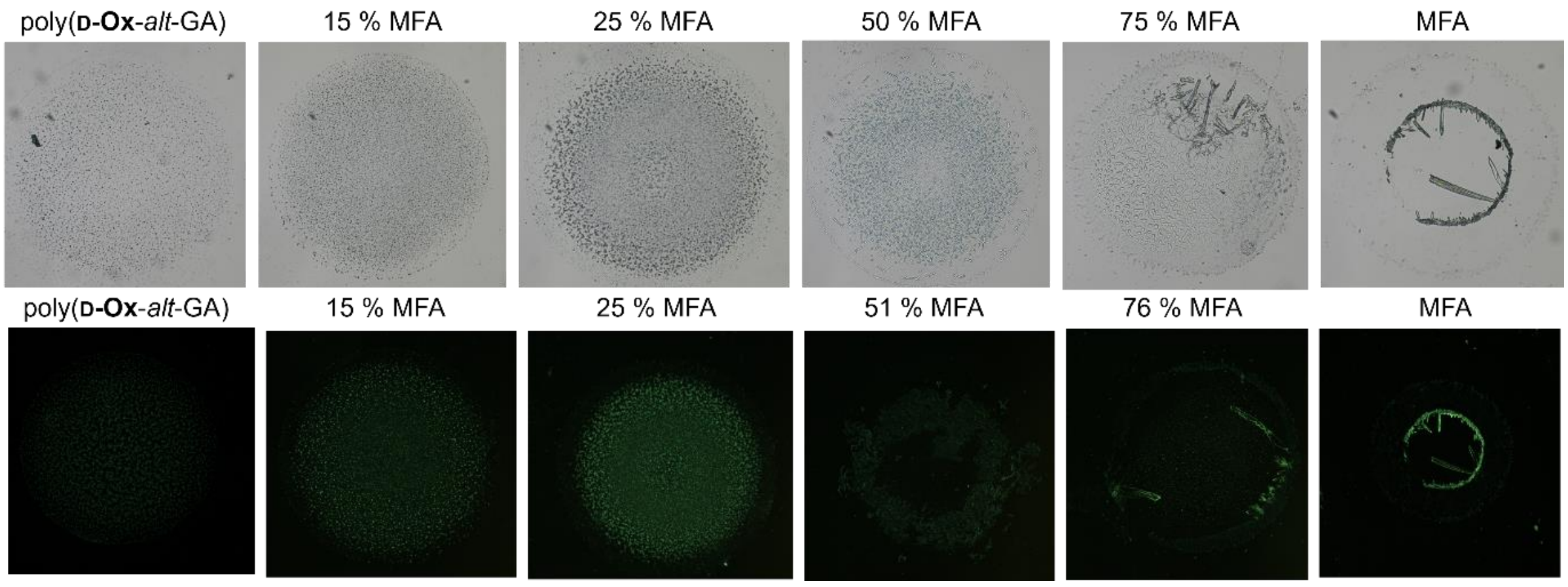

Figure S5. Optical microscopy(top) and polarised optical microscopy (bottom) images of the poly(**D-Ox**-*alt*-GA) MFA microarray varying the weight/weight ratio from 0 – 100 % (two days after printing).

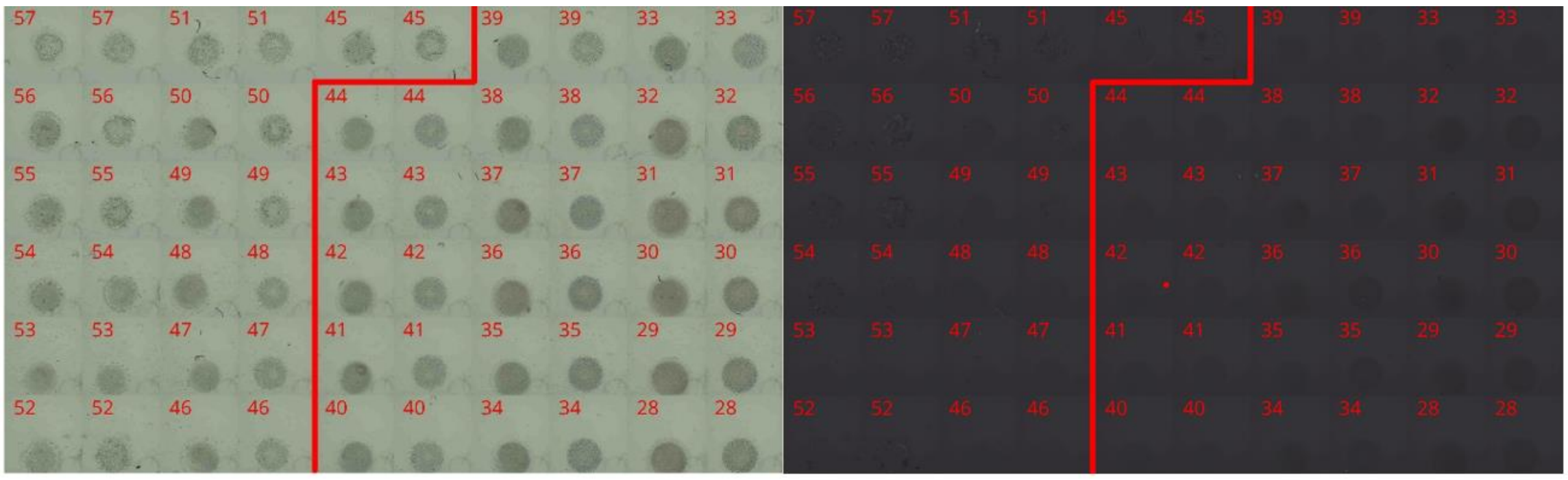

Figure S6. Phase contrast (left) and cross polar (right) microscopy images (two months after printing) for % w/w combinations (as labelled) of MFA with poly(**D-Ox**-alt-GA). Each individual image is ca. 1.75 mm across. Columns are in duplicate: the drug-first printed spots appear on the left and the excipient-first on the right. Numbers refer to the loading of MFA (% w/w). The red line denotes the estimated transition point where birefringence appears (as a function of increasing composition).

4.2 DSC traces and FTIR spectra of Nifedipine ASDs

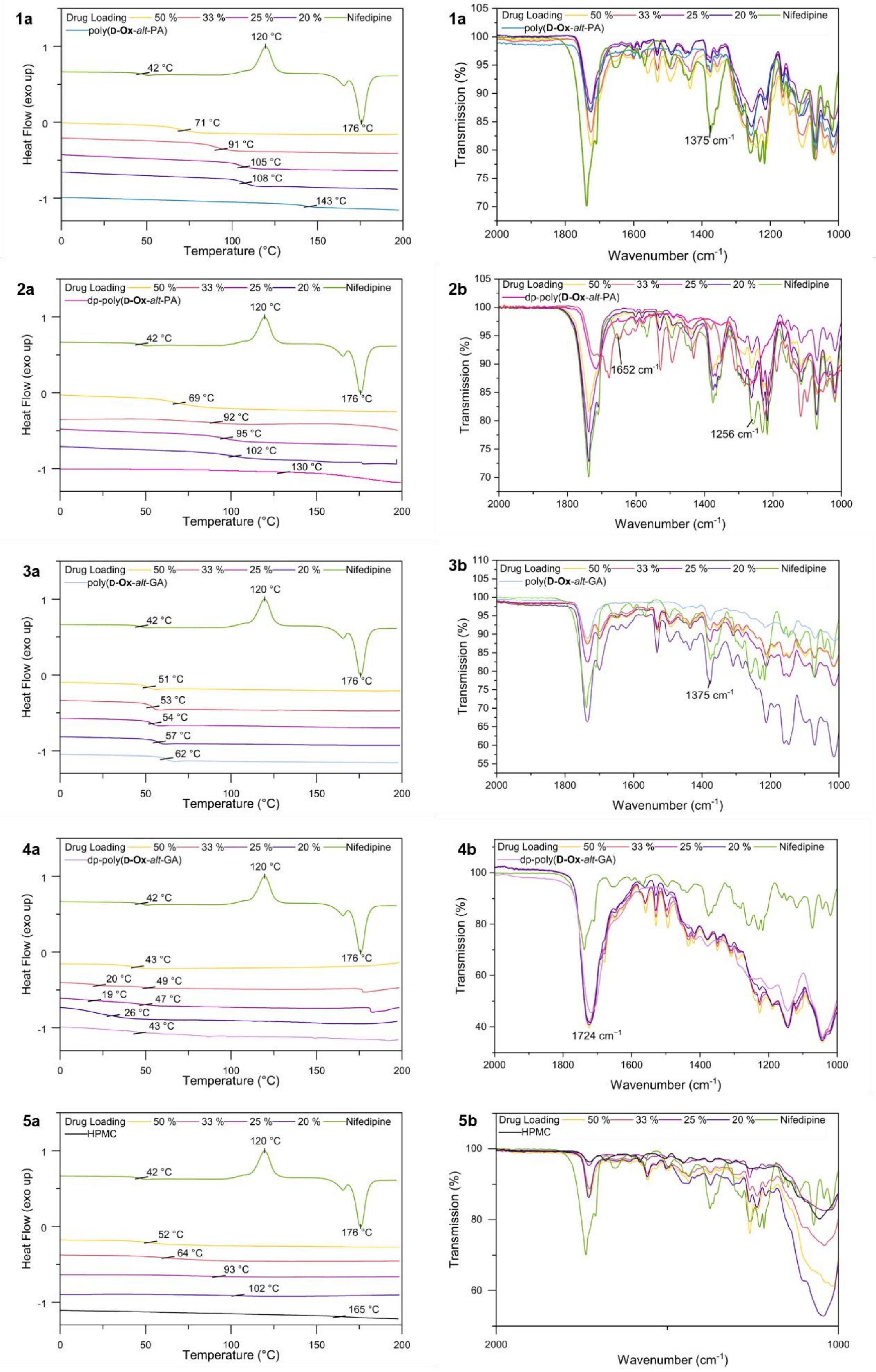

Figure S7. DSC traces (exo up, second cooling cycle), 20 °C min^{-1} (a) and FTIR spectra between 2000 and 1000 cm^{-1} (b) of Nifedipine ASDs with (1) poly(**D-Ox**-*alt*-PA), (2) dp-poly(**D-Ox**-*alt*-PA), (3) poly(**D-Ox**-*alt*-GA), (4) dp-poly(**D-Ox**-*alt*-GA) and (5) HPMC.

4.3 DSC traces, PXRD patterns and FTIR spectra of MFA ASDs

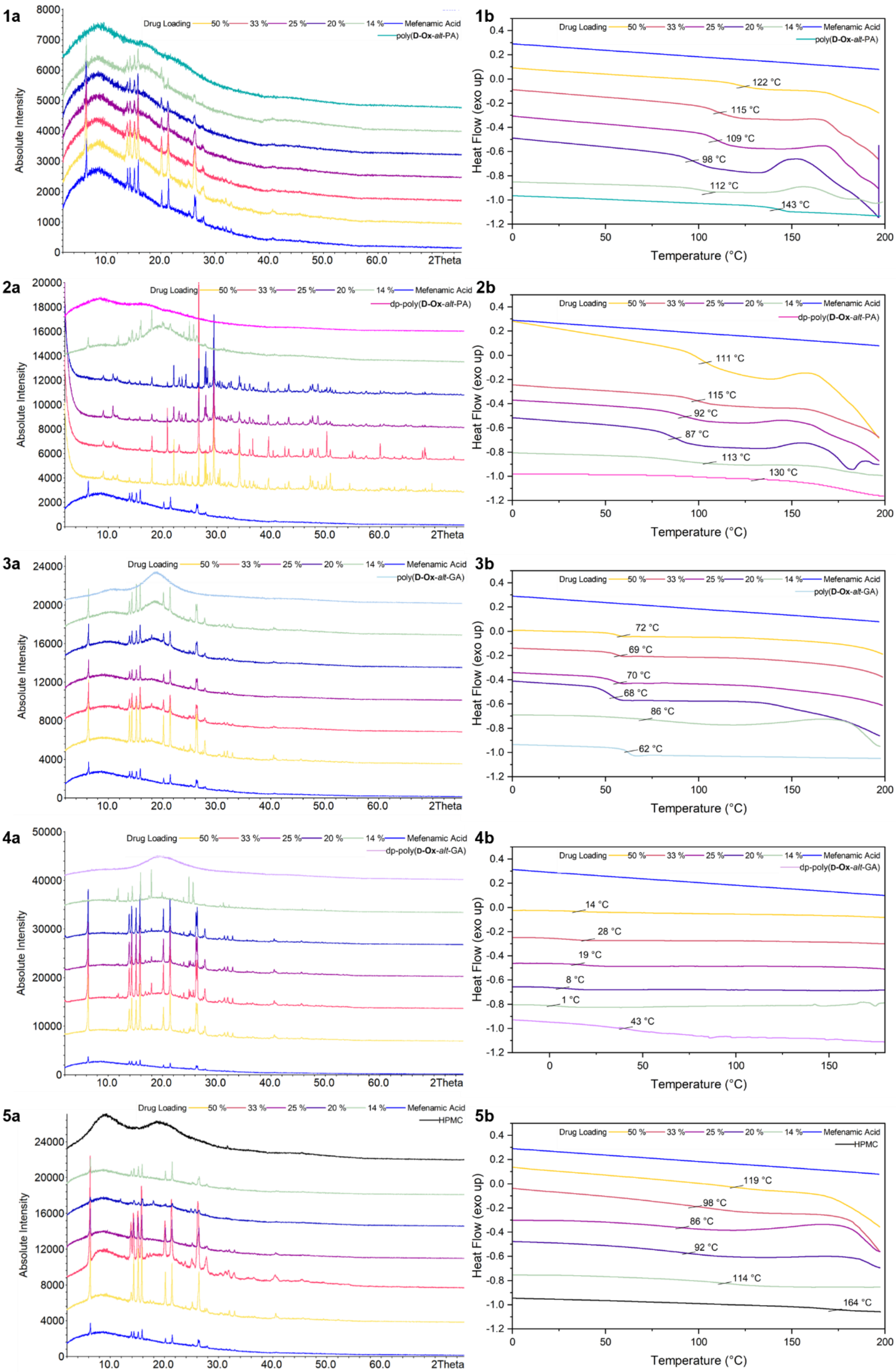

Figure S8. PXRD patterns (a) and DSC traces (exo up, second cooling cycle), 20 °C min^{-1} (b) of MFA ASDs with (1) poly(**D-Ox**-*alt*-PA), (2) dp-poly(**D-Ox**-*alt*-PA), (3) poly(**D-Ox**-*alt*-GA), (4) dp-poly(**D-Ox**-*alt*-GA) and (5) HPMC.

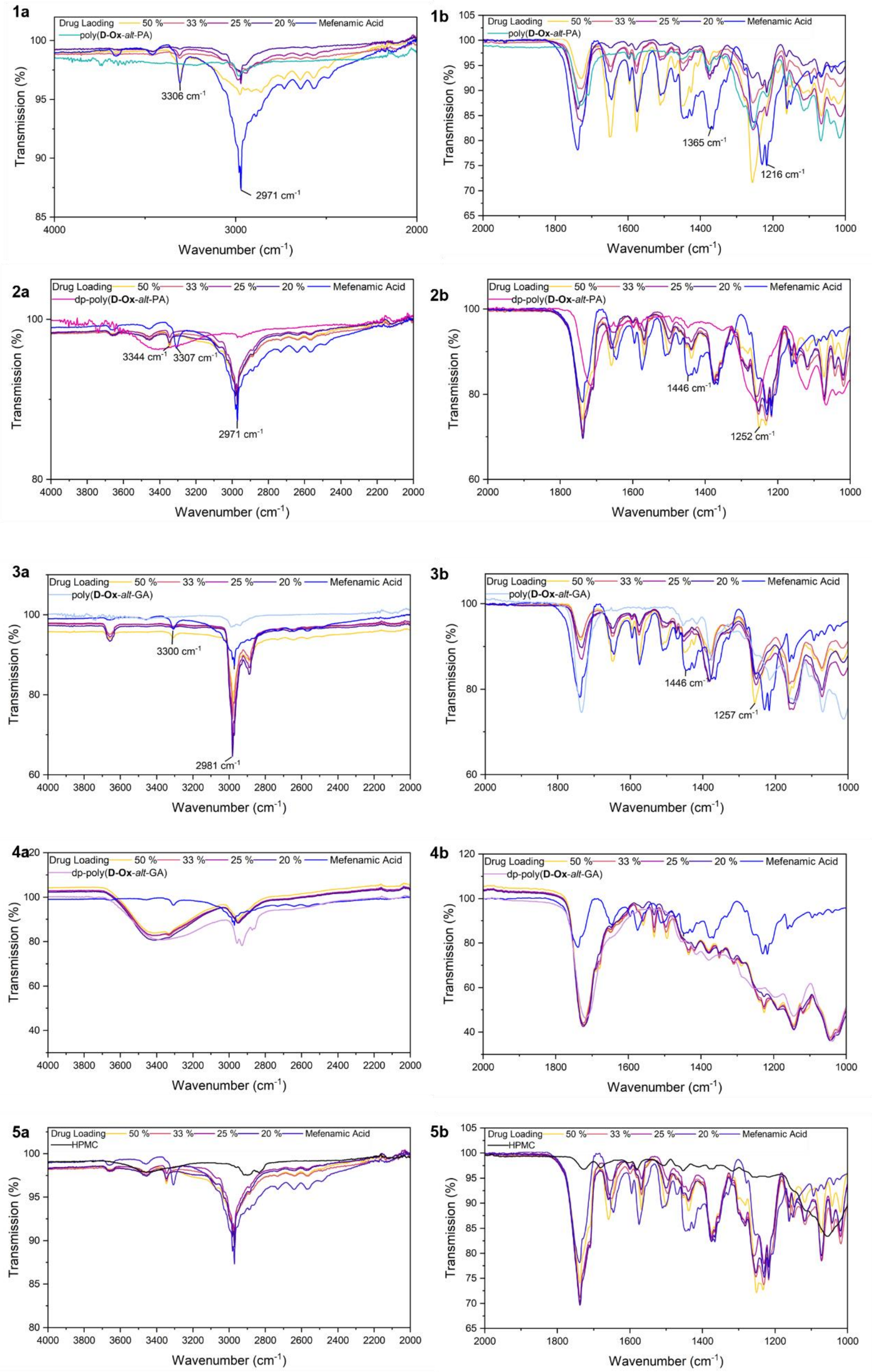

Figure S9. FTIR spectra between 2000 and 1000 cm^{-1} (a) and 4000 and 2000 cm^{-1} (b) of MFA ASDs with (1) poly(**D-Ox**-*alt*-PA), (2) dp-poly(**D-Ox**-*alt*-PA), (3) poly(**D-Ox**-*alt*-GA), (4) dp-poly(**D-Ox**-*alt*-GA) and (5) HPMC.

4.4 UV-vis spectra

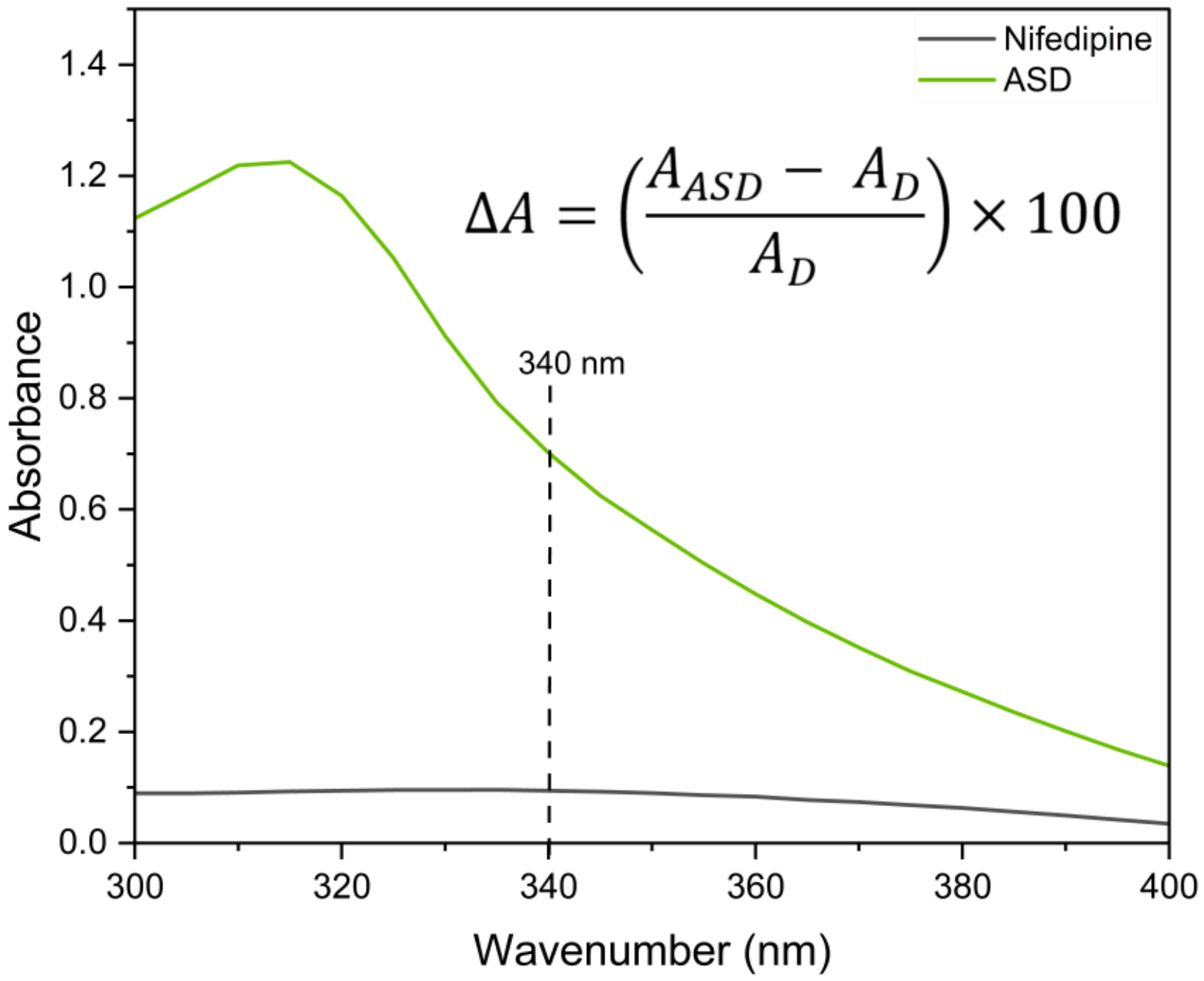

Figure S10. Example UV-vis spectra for ΔA calculations for Nifedipine ASDs, green spectra is the HMPC–Nifedipine ASD absorbance spectra, and the black is the free Nifedipine absorbance spectra

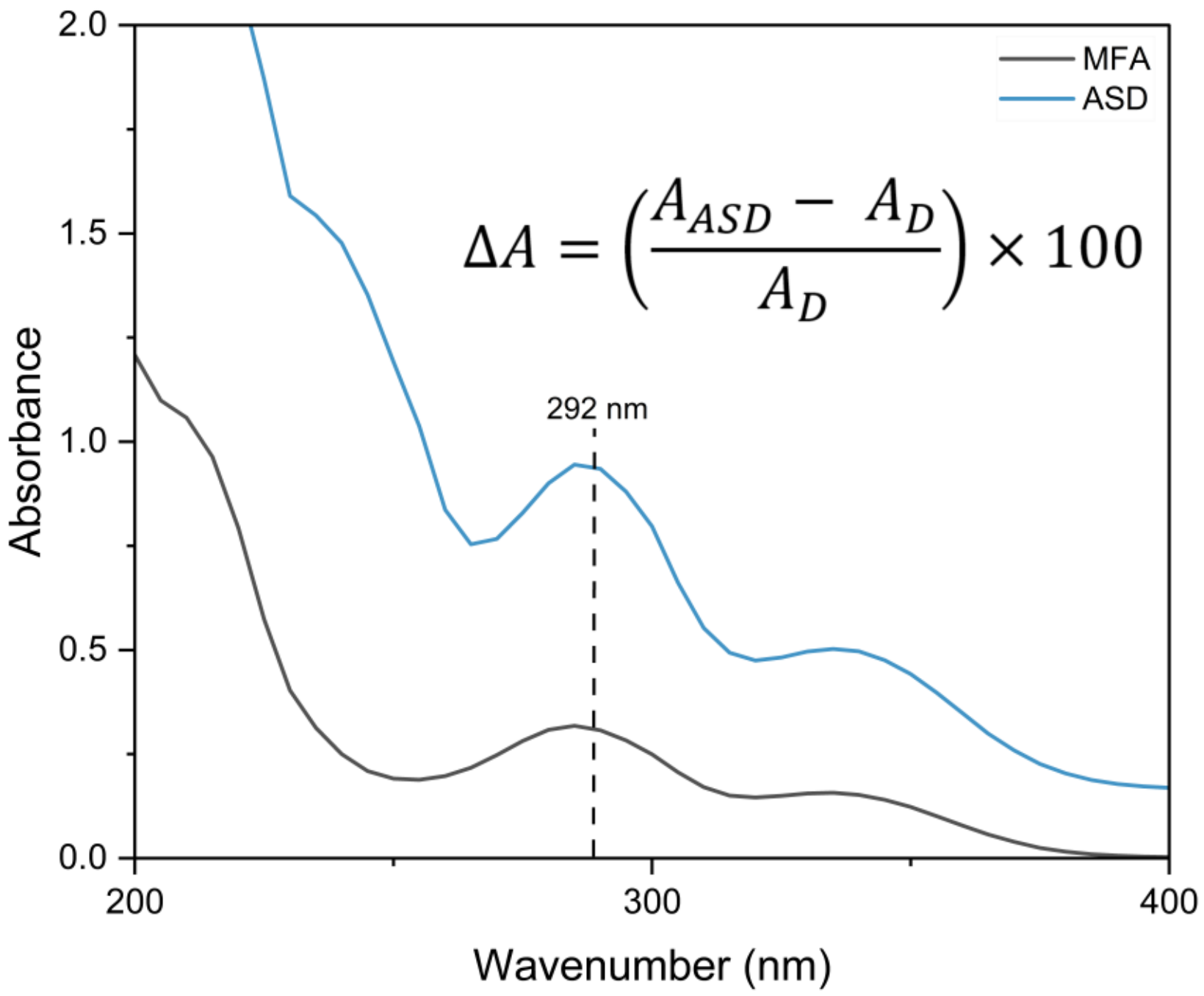

Figure S11. Example UV-vis spectra for ΔA calculations for MFA ASDs, blue spectra is the HMPC–MFA ASD absorbance spectra, and the black is the free MFA absorbance spectra.

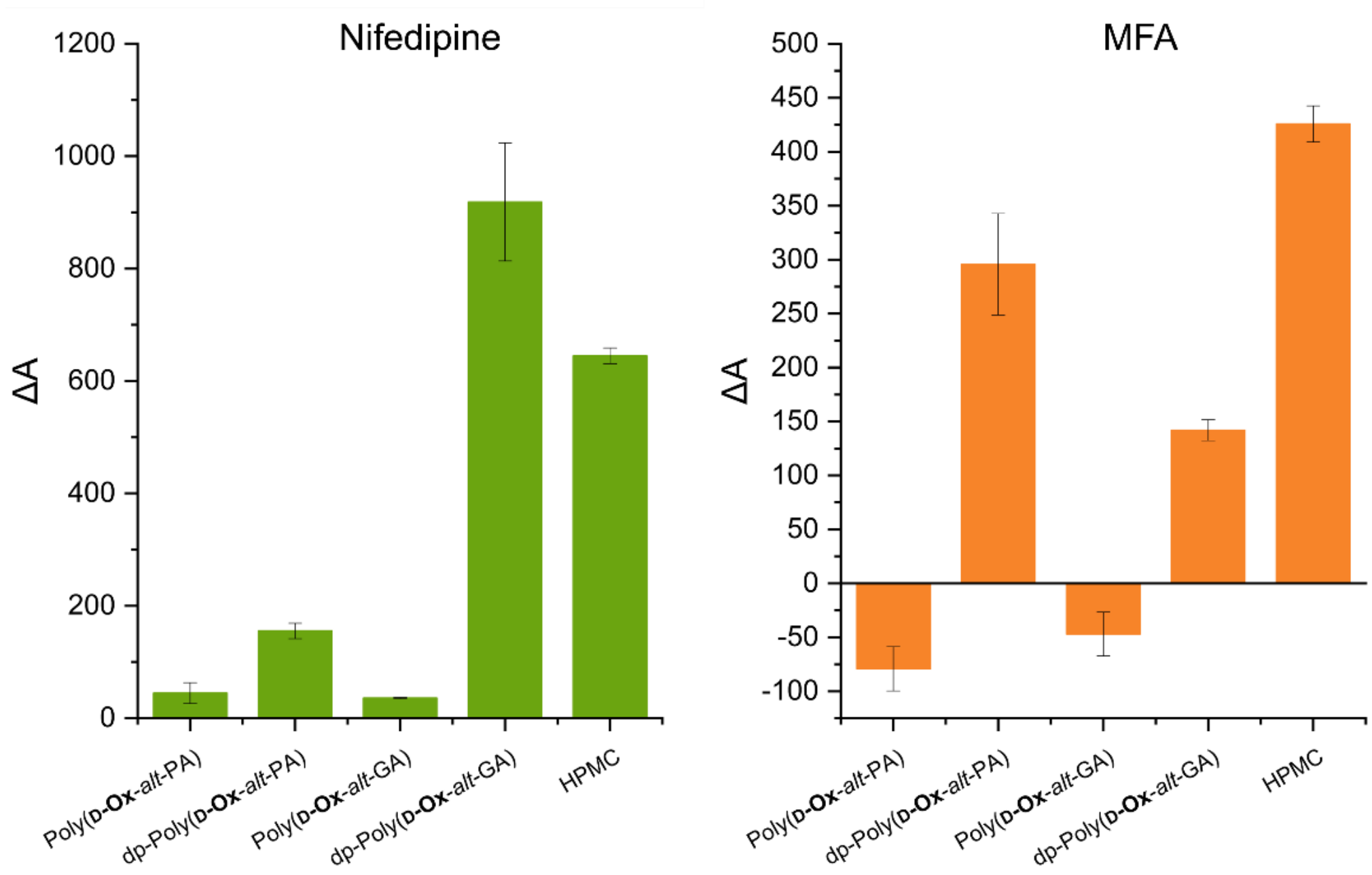

Figure S12. Average ΔA value for ASDs with Nifedipine and MFA showing all tested polymers.

4.5 Computational Modelling

Table S2. Data from molecular dynamics simulations for modelled 20-mer (assume full deprotection).

**Polymer**	**Calculated solubility parameter ((MJ/m^3)^{½})**
Nifedipine	22.1
MFA	18.5
dp-poly(**D-Ox**-*alt*-GA) (20mer)	11.7
dp-poly(**D-Ox**-*alt*-PA) (20mer)	19.0

4.6 Summary table

Table S3 Summary of data collected on various ASDs.

Drug	Excipient	Highest amorphous composition after 3 days [after 2 months] (% w/w)[a]	Highest amorphous composition after 3 days (% w/w)[b]	ΔT_g[c] (°C)	T_g at HAC[d] (°C)	Solubility parameters MFA minus excipient ($(MJ/m^3)^{½}$)	ΔA[e]
Nifedipine	dp-poly(**D-Ox**-*alt*-GA)	Amorphous	50	+1	43	-	918
	dp-poly(**D-Ox**-*alt*-PA)	Amorphous	50	+88	69	-	155
	poly(**D-Ox**-*alt*-GA)	Amorphous	50	+20	51	-	35
	poly(**D-Ox**-*alt*-PA)	-	50	+101	71	-	45
	HPMC	-	-	+123	102	-	645
MFA	dp-poly(**D-Ox**-*alt*-GA)	25 [14]	<14	-8	1	-0.5	142
	dp-poly(**D-Ox**-*alt*-PA)	38 [38]	<14	+79	113	6.8	296
	poly(**D-Ox**-*alt*-GA)	51 [44]	<14	+11	86	-	-47
	poly(**D-Ox**-*alt*-PA)	-	<14	+92	112	-	-79
	HPMC	-	<14	+113	114	-	426

[a] Microarray data. [b] DSC/PXRD data of upscaled ASDs. [c] Difference in T_g between drug and polymer. [d] T_g at the highest amorphous composition in upscaled ASDs. [e] ΔA value after two hours in water (drug solubility enhancement when in ASD compared to free drug).
